# Targeting Cpt1a-Bcl-2 interaction modulates apoptosis resistance and fibrotic remodeling

**DOI:** 10.1038/s41418-021-00840-w

**Published:** 2021-08-20

**Authors:** Linlin Gu, Ranu Surolia, Jennifer L. Larson-Casey, Chao He, Dana Davis, Jungsoon Kang, Veena B. Antony, A. Brent Carter

**Affiliations:** 1 Department of Medicine, Division of Pulmonary, Allergy, and Critical Care Medicine, University of Alabama at Birmingham, Birmingham, AL USA; 2 Birmingham VAMC, Birmingham, AL USA

**Keywords:** Cell biology, Medical research

## Abstract

The mitochondrial calcium uniporter (MCU) regulates metabolic reprogramming in lung macrophages and the progression of pulmonary fibrosis. Fibrosis progression is associated with apoptosis resistance in lung macrophages; however, the mechanism(s) by which apoptosis resistance occurs is poorly understood. Here, we found a marked increase in mitochondrial B-cell lymphoma-2 (Bcl-2) in lung macrophages from subjects with idiopathic pulmonary fibrosis (IPF). Similar findings were seen in bleomycin-injured wild-type (*WT*) mice, whereas Bcl-2 was markedly decreased in mice expressing a dominant-negative mitochondrial calcium uniporter (*DN-MCU*). Carnitine palmitoyltransferase 1a (Cpt1a), the rate-limiting enzyme for fatty acid β-oxidation, directly interacted with Bcl-2 by binding to its BH3 domain, which anchored Bcl-2 in the mitochondria to attenuate apoptosis. This interaction was dependent on Cpt1a activity. Lung macrophages from IPF subjects had a direct correlation between CPT1A and Bcl-2, whereas the absence of binding induced apoptosis. The deletion of Bcl-2 in macrophages protected mice from developing pulmonary fibrosis. Moreover, mice had resolution when Bcl-2 was deleted or was inhibited with ABT-199 after fibrosis was established. These observations implicate an interplay between macrophage fatty acid β-oxidation, apoptosis resistance, and dysregulated fibrotic remodeling.

## Introduction

Pulmonary fibrosis is a chronic disease that consists of aberrant remodeling of lung tissue. Idiopathic pulmonary fibrosis (IPF) is the most common form of pulmonary fibrosis and has a high mortality rate within 3–5 years after diagnosis [1]. The currently approved medications for IPF have limited efficacy based on the absence of changes in quality of life or mortality [2, 3]. Thus, understanding the cellular and molecular mechanisms in the pathogenesis of IPF may lead to more effective therapies.

Both apoptosis and resistance to apoptosis are associated with fibrosis in multiple organ systems [4–6]. Increased alveolar epithelial cell (AEC) injury and apoptosis are proposed to be the initiating event in lung fibrosis [1]. In contrast, apoptosis resistance in fibroblasts is critical for aberrant lung remodeling in IPF [7]. Macrophages in chronic disease exhibit apoptosis resistance, and their prolonged survival is generally associated with disease progression [8, 9]. Moreover, conditional macrophage depletion attenuates models of liver and lung injury in vivo [10, 11]. Although prior data demonstrated cleaved caspase-3 was reduced in lung macrophages from IPF subjects [9], the exact mechanism by which this occurs has not been determined.

The B-cell lymphoma-2 (Bcl-2) family of proteins are critical regulators of programmed cell death. The pro-apoptotic Bid gene in AECs [12] and Bcl-2 expression in myofibroblasts are key factors in fibrosis development [13]. The pharmacologic inhibition of Bcl-2 reversed established skin fibrosis by inducing apoptosis of myofibroblasts in vitro [14]. Based on the decisive role of lung macrophages in orchestrating fibrosis development and progression [15, 16], the molecular mechanism(s) by which Bcl-2 is regulated in lung macrophages is critical to understand the pathogenesis of dysregulated fibrotic remodeling.

Metabolic reprogramming that entails fatty acid β-oxidation (FAO) and oxidative phosphorylation is a feature in macrophage activation in multiple diseases [17, 18]. The mitochondrial calcium uniporter (MCU) is known to enhance aerobic glycolysis in cancer [19, 20]; however, we recently showed that MCU regulated metabolic reprogramming to FAO, in part, by increasing expression and activity of Cpt1a [21]. Inhibition of Cpt1a and FAO pharmacologically has been used as a potential therapeutic by inducing apoptosis in breast cancer cells. The mechanism(s) by which the drug-induced apoptosis occurs has not been determined at the molecular level [22]. Prior evidence showed that apoptosis resistance in lung macrophages occurs in fibrosis development [9], but it is not known if MCU regulation of Cpt1a has a direct role in mediating apoptosis resistance in lung macrophages or if this regulation has an impact on the progression of fibrotic remodeling.

## Materials/Subjects and methods

### Human subjects

The Institutional Review Boards of the University of Alabama at Birmingham and the Birmingham VAMC approved the protocols of obtaining lung macrophages from normal and IPF subjects. Informed consent was obtained from all participating subjects. Normal subjects had to meet the following criteria: (1) age between 30 and 75 years old; (2) no history of cardiopulmonary disease or other chronic disease; (3) no prescription or non-prescription medication except oral contraceptives; (4) no recent or current evidence of infection; and (5) lifetime nonsmoker. The IPF subjects (Table 1) had to meet the following criteria: (1) forced vital capacity had to be at least 50% predicted; (2) current nonsmoker; (3) no recent or current evidence of infection; (4) evidence of restrictive physiology on pulmonary function tests; and (5) usual interstitial pneumonia pattern on high-resolution chest computed tomography scans. Fiberoptic bronchoscopy with bronchoalveolar lavage (BAL) was performed. Three subsegments of the lung were lavaged with five 20-ml aliquots of normal saline, and the first aliquot in each was discarded. The percentage of lung macrophages determined by Wright-Giemsa stain was 90–98%.

**Table 1:** Patient demographics.

IPF subjects							
Male				Female			
Age	FVC	FVC%	DLCO%	Age	FVC	FVC%	DLCO%
70	3.86	106%	57%	76	2.19	72%	54%
64	2.85	60%	56%	74	3.17	113%	43%
77	2.66	59%	44%	78	2.54	83%	55%
74	2.80	68%	44%				
65	2.60	53%	51%				
72	2.59	73%	68%				

*FVC* forced vital capacity, *FVC%* percent predicted, *DLCO* diffusion capacity for carbon dioxide percent predicted.

### Mice

All protocols were approved by the University of Alabama at Birmingham Institutional Animal Care and Use Committee. *DN-MCU-Lyz2-cre* mice and their WT littermates were described previously [21]. *Bcl-2*^*tm1lrt*^*/J* mice (Jackson Laboratory# 008882) were crossed with *Cre*^*TAM*^*-Csf1r* mice to generate *Bcl2*^*−/−*^*Csf1r*^*MeriCreMer*^ mice and their *Bcl2*^*fl/fl*^ littermates. *Bcl2*^*−/−*^*Csf1r*^*MeriCreMer*^ mice, unless otherwise stated, were fed tamoxifen-containing chow (Envigo# TD.130860) for 2 weeks prior to bleomycin exposure. Alternatively, *Bcl2*^*−/−*^*Csf1r*^*MeriCreMer*^ mice were administered corn oil dissolved tamoxifen or corn oil for 8 days (20 mg/kg i.p.), starting from day 12 after exposure. Male and female mice, 6–12 weeks old, were administered bleomycin (1.75 U/kg) or sterile saline intratracheally. In separate experiments, mice were administered chrysotile asbestos (100 μg) or man-made vitreous fiber, as a control. For each independent mice experiment, a minimum of five mice of certain genotype were randomly allocated into each experimental group, in favor of statistical analysis. The pre-established exclusion criteria are: (1) mice younger than 6 weeks old and older than 12 weeks old; (2) mice above the average body weight; and (3) mice with obvious disabilities. Investigators were blinded to genotype during experiments and/or when assessing the outcome.

### In vivo treatment of ABT-199

ABT-199 (ApexBio #A8194) was reconstituted in the solution that contains 10% ethanol, 30% polyethylene glycol 400 (PEG 400), and 60% phosal 50 propylene glycol. At day 12 after bleomycin exposure, mice were administered ABT-199 or vehicle (50 mg/kg) via oral gavage daily through day 21.

### Mitochondrial permeability transition pore opening

The pore opening was analyzed with MitoProbe Transition Pore Assay Kit (Molecular Probes #M34153), according to the manufacturer’s instructions.

### Respiratory mechanics analysis

Mice were anesthetized using xylazine (5 mg/kg) and ketamine (130 mg/kg), and tracheotomy for intubation was performed. Mice were connected to a computer-controlled mechanical ventilator (FlexiVent, SCIREO, Montreal, Canada) via the Y-tubing supported by FlexiWare 8 software and mechanically ventilated at 150 breaths/min, tidal volume of 10 ml/kg, and a positive end-expiratory pressure of 3 cmH_2_O. ECG was recorded continuously to monitor the heart rate. Mice were paralyzed with 1.0 mg/kg of pancuronium bromide (Hospira). Deep inflation perturbation (27 cmH_2_O) was performed to verify the cannula insertion and attachment, and PVs-P (27 cmH_2_O) protocol was applied to confirm the absence of spontaneous inspiratory efforts. The scripts used in the current study included SnapShot-150 (10 ml/kg), Quick Prime3 (3 ml/kg). Compliance (Cst) was evaluated using the PV-Loop Salazar Knowles equation. Tissue stiffness (H) was measured by applying the constant-phase model. Investigators were blinded to genotype during experiments and when assessing the outcome.

### Plasmids, small interfering RNAs, and transfections

pCDNA3.1-Bcl-2 was generated by ligating mouse Bcl-2 gene open reading frame (ORF) into pcDNA™3.1/V5-His TOPO vector (Invitrogen# K480001). The Bcl-2 ORF was amplified by PCR using cDNA template. Truncations (ΔBH1, ΔBH2, ΔBH3, and ΔBH4) of Bcl-2 plasmid were derived from pCDNA3.1-Bcl-2 by site-directed deletion using Q5 Site-Directed Mutagenesis Kit (NEB# E0552S). pCDNA3.1-MCU_WT_, pCDNA3.1-MCU_DN_, mouse MCU shRNA plasmids, and pLKO.1 puro were described previously [21]. Mouse Cpt1a shRNA plasmid was purchased from Sigma-Aldrich (TRCN0000325593 in #SHCLNG-NM_013495). Plasmids were transfected with X-treme Gene 9 Transfection Reagent (Roche# 06365809001), according to the manufacturer’s instructions. After 24–48 h, cells were subjected to either treatment of specific interest or collected for downstream purposes. Mouse Bcl-2 siRNA and human Bcl-2 siRNA were purchased from IDT (Coralville, IA) and transfected using DharmaFECT 4 (Dharmacon# T-2004) or DharmaFECT 2 (Dharmacon# T-2002), according to the manufacturer’s instructions. Co-transfections of siRNA with a plasmid at the same time were performed using DharmaFECT Duo (Dharmacon# T-2010).

### Cell culture

Human THP-1 monocyte and mouse MH-S alveolar macrophage cell lines were purchased from American Type Culture Collection with the certificate of authentication and mycoplasma negative test, and cells were maintained as described previously [23].

### Mitochondria and nuclei isolation

Mitochondria, nuclei, and cytoplasm were isolated as previously described [24].

### Immunoprecipitation and Purification of V5-His-tagged protein

For immunoprecipitation, at least 10 million cells were lysed in NP40 lysis buffer supplemented with EDTA-free protease inhibitor cocktail. Supernatant from centrifugation was filtered (0.22 μm). Beads from Dynabeads Protein G Kit (Invitrogen# 10007D) were incubated with primary antibodies Cpt1a (ProteinTech# 15184-1-AP) to form beads-antibody complex. To avoid co-elution of the bound antibody, the complex was crosslinked with disuccinimidyl suberate (Thermo Scientific# A39267), according to the manufacturer’s instructions. Equal amount of total protein from supernatant was incubated with the beads-antibody complex for 1 h at room temperature. The incubation complex was then washed three times and eluted. Purification of V5-His-tagged proteins was performed as previously described [25].

### Immunoblot analysis

Primary antibodies used in immunoblot analysis were as follows: MCU (D2Z3B) rabbit mAb (CellSignaling# 14997); Bcl-2 rabbit pAb (ABclonal# A2845); Cpt1a mouse mAb (Abcam# 128568); Caspase-3(8G10) rabbit mAb (Cell Signaling# 9665); Caspase-9(C9) mouse mAb (Cell Signaling# 9508); Caspase-8 rabbit pAb (R&D Systems# af1650); Bax rabbit Ab (CellSignaling# 2772 S), Bak rabbit pAb (ABclonal# A0204); Puma rabbit pAb (ABclonal# A17138); anti-Noxa rabbit pAb (ABclonal# A9801); Cytochrome c mouse mAb (SantaCruz#sc-13156); VDAC rabbit pAb (CellSignaling# 4866); Lamin A/C rabbit pAb (CellSignaling# 2032); TGF-β1 mouse mAb (R&D Systems# MAB240); and β-actin mouse mAb (Sigma-Aldrich# A5441). All antibodies were 1:1000 diluted except the β-actin mouse mAb diluted at 1:10,000. Results were quantified using Image J.

### TUNEL assay

Cells on cytospin slides were fixed in 4% paraformaldehyde in PBS for 30 min. Cells were rinsed in PBS and permeabilized in ice-cold permeabilization solution (0.1% sodium citrate and 0.1% Triton X-100 in ddw) for 2 min. Cells were subjected to TUNEL staining using the In-Situ Cell Death Detection Kit, TMR Red (Roche # 12156792910), according to the manufacturer’s instructions. Cells were counterstained with DAPI. TUNEL staining was evaluated by confocal analysis, and data were represented as TUNEL density over the corresponding DAPI area. For co-staining TUNEL with CD206-FITC, cells were stained with TUNEL first, followed by CD206-FITC staining.

### Caspase-3 activity measurement

Caspase-3 activity was quantitated with the EnzCheck® Caspase-3 Assay Kit #2 (Molucular Probes# MP13184). Cells were lysed for 30 min on ice in cell lysis buffer, and centrifuged (5000 rpm × 10 min, 4 °C). Equal amount of protein from supernatant was distributed to each well of a 96-well black plate. Reaction buffer (1x) containing a final concentration of 50 μM Z-DEVD-R110 (CPC scientific# CASP-066B) was mixed into the wells. The plate was subjected to a kinetic reading, as described in the manufacturer’s instructions.

### Flow cytometry-based annexin V measurement

The Dead Cell Apoptosis Kit (Thermo Fisher# V13242) was used to quantitate annexin V-positive live cells, according to the manufacturer’s instructions. Before the Annexin V-FITC and PI staining, BAL cells were blocked and stained with antibody cocktails to distinguish monocyte-derived macrophages (MDM) from tissue-resident macrophages (resident alveolar macrophage (RAM)), as described previously [15, 16].

### Indirect fluorescence assay

Cells were fixed with 4% formaldehyde at room temperature for 45 min, followed by permeabilization for 3 min in ice-cold buffer (0.1% sodium citrate and 0.1% Triton X-100 in distilled water). Cells were blocked at room temperature for 1 h in DPBS with 1% BSA, and then incubated with Cpt1a mouse mAb (Abcam# ab128568), Bcl-2 rabbit pAb (ABclonal# A2845), Noxa rabbit pAb (ABclonal# A9801), or Puma rabbit pAb (ABclonal# A17138). All images o were quantitated using Image J. All data were calculated as the intensity units arbitrary to corresponding DAPI intensity or as fold change to control.

For confocal analysis of colocalization between MitoTracker Red and Bcl-2, cells were first stained with MitoTracker Red (250 nM, 20 min), and then fixed with stained with Bcl-2 antibody, as described above.

### Fatty acid oxidation by oxygen consumption rate (OCR) determination

OCR measurement by a Seahorse XF96 bioanalyzer (Seahorse Bioscience) was performed as described [21].

### ELISA

Active TGF-β1, TNF-α, and MCP-1 in BAL fluid from mice or conditioned media were measured by using ELISA (R&D Systems# DY1679 for TGF-β1, DY410 for TNF-α), according to the manufacturer’s instructions.

### Hydroxyproline assay

Hydroxyproline in lung tissue was measured as previously described [26].

### Cpt1a activity measurement

Cpt1a activity was measured as previously described [21].

### Real-time quantitative PCR

Total RNA was isolated using Trizol reagent (Thermo Fisher Scientific#15596018) and reverse transcribed with iScript reverse transcription kit (Bio-Rad# 170-8891). Expression of mRNA was determined by real-time quantitative PCR using the SYBR Green Kit (Bio-Rad# 170-8882). Data were calculated by using the ^∆∆^C_*t*_ method. Measurements were normalized to β-actin (mouse) and expressed in arbitrary units.

### IHC-P

IHC-P detection of α-SMA by chromogenic staining was described previously [21]. IHC-P detection of α-SMA, Vimentin, and TUNEL by immunofluorescent staining was performed as described elsewhere [16]. Antibodies used include α-SMA (American Research Products #03-61001) and Vimentin (Abclonal #A19607).

### Mouse type II alveolar epithelial cells (AEC) isolation

Mice lungs were rinsed in sterile 1x PBS, immersed in 3 ml Dispase (add 20 μl DNase I (20,000 U/ml) to 20 ml Dispase; filtered at 0.22 μm), and minced with scissors to 1 mm pieces. The minced pieces were transferred to an 50 ml-erlenmeyer flask and incubated with magnetic stirring for 2 h at 37 °C. Overall, 25 ml DMEM supplemented with 10% carbon-stripped FBS, HEPES, Gentamicin, and Amphotericin B was added to the same flask. The whole suspension was poured through sterile gauzes, 100 and 20 μM filters, and centrifuged for 10 min at 1000 rpm. The resulting cell pellet was resuspended in the DMEM without carbon-stripped FBS, transferred to petri dishes coated with IgG, and incubated for hour in culture incubator. The cells removed from the IgG-coated dishes are the Type II AEC confirmed by immunoblot analysis for SPC.

### Statistical analyses

Statistical analyses were performed using Graphpad Prism 5 statistical software. A minimum of three biological replicates were required in determining the statistical significance. Considering the variability of human samples, a minimum of five biological replicates were required for statistical calculation. All data were expressed as mean ± SEM. Normal distribution was analyzed to determine whether data met the assumptions of the statistical test. Groups with similar variances were used for statistical comparisons. Statistical analyses were performed with either an unpaired two-sided student’s *t*-test, one-way ANOVA with Tukey’s post hoc test, or two-way ANOVA with *p* < 0.05 considered significant.

## Results

### Macrophage MCU regulates apoptosis resistance in lung macrophages by increasing Bcl-2

**Fig. 1:**
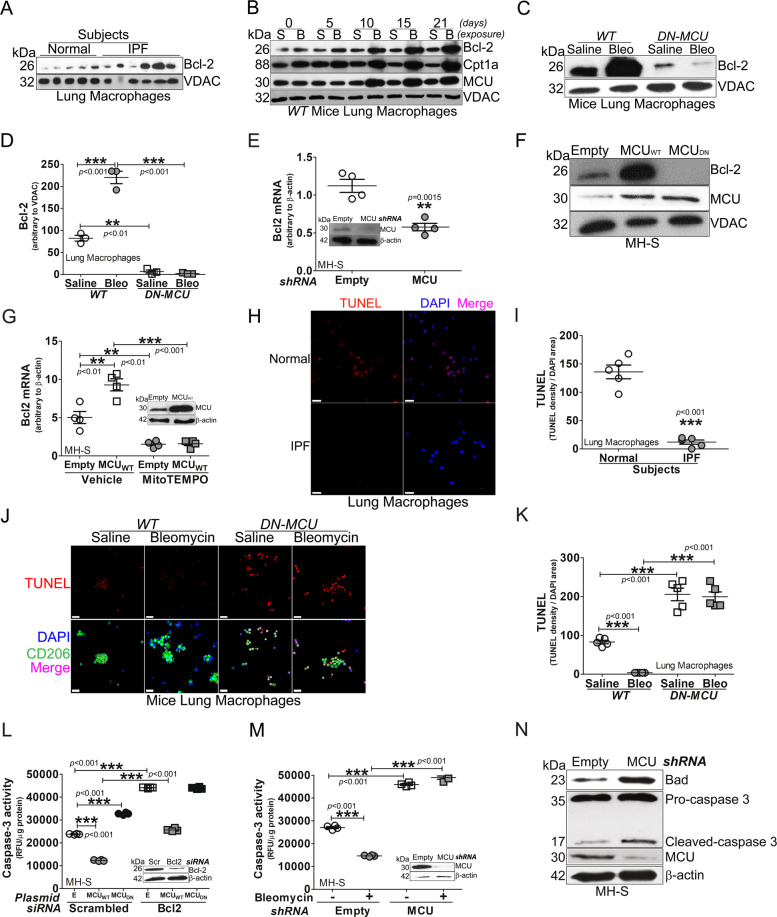
Macrophage MCU regulates apoptosis resistance in lung macrophages by increasing Bcl-2. **A** Lung macrophages from subjects (IPF or normal) were subjected to mitochondrial isolation and detection of Bcl-2 by immunoblot analysis. **B** Lung macrophages from WT mice exposed to bleomycin or saline control were subjected to mitochondrial isolation and detection of Bcl-2, Cpt1a, and MCU by immunoblot analysis. **C** *DN-MCU-Lyz2-cre* mice and *WT* littermates were exposed to saline or bleomycin. Lung macrophages were isolated after 21 days and subjected to mitochondrial isolation and detection of Bcl-2 by immunoblot analysis. *DN-MCU* (*DN-MCU-Lyz2-cre*). **D** Mitochondrial Bcl-2 in C was quantified and normalized to VDAC, *n* = 3. **E** MH-S cells were transfected to silence MCU. Total RNA was extracted for the determination of Bcl2 mRNA by quantitative real-time RT-PCR (qRT-PCR), *n* = 4. **F** MH-S cells were transfected with MCU_WT_, MCU_DN_, or empty control. Mitochondria were isolated for the detection of Bcl-2 and MCU by immunoblot analysis. **G** MH-S cells were transfected to overexpress MCU_WT_ and treated with MitoTEMPO (50 μM, overnight). Bcl2 mRNA was determined by qRT-PCR, *n* = 4. BAL cells from normal or IPF subjects were stained with TUNEL. The staining was **H** imaged by confocal microscopy, scale bars at 20 μm, and **I** statistically quantitated, *n* = 5. BAL macrophages from bleomycin- or saline-exposed *DN-MCU-Lyz2-cre* mice or *WT* littermates were stained with TUNEL and a macrophage marker, CD206. The staining was **J** imaged by confocal microscopy, scale bars at 20 μm, and **K** statistically imaged, *n* = 5. **L** MH-S cells were co-transfected with empty, MCU_WT_, or MCU_DN_ with scrambled or Bcl-2 siRNA. Caspase-3 activity was measured, *n* = 4. E, empty; Scr, scrambled siRNA. **M** MH-S cells were transfected to empty or MCU shRNA and exposed to bleomycin (0.0126 U/ml; 1 h). Caspase-3 activity was measured, *n* = 4. Inset: Immunoblot analysis for MCU. **N** MH-S cells were transfected with empty or MCU shRNA. Immunoblot analysis for Bad, caspase-3, and MCU was performed. One-way ANOVA with Tukey’s post hoc comparison. Two-tailed student’s *t*-test (**E**, **I**). *p ≤ 0.05, **p ≤ 0.01, and ***p ≤ 0.001. See also Fig. S1.

Prior evidence showed that the active caspase-3 was markedly reduced in lung macrophages from IPF subjects [9], but the mechanism(s) by which it is reduced is not known. We found that Bcl-2 expression was nearly fourfold higher in lung macrophage mitochondria from IPF subjects compared to normal subjects (Figs. 1A and S1A). Bcl-2 expression increased at day 10 in lung macrophage mitochondria from bleomycin-injured mice and remained elevated through 21 days (Fig. 1B). MCU and Cpt1a had a similar increase in mitochondrial expression.

Because Bcl-2 and MCU had a similar expression in response to bleomycin, we asked if MCU regulated the expression of Bcl-2. Bcl-2 was significantly increased in bleomycin-injured *WT* mice, whereas expression in lung macrophages from *DN-MCU-Lyz2-cre* mice was less than the saline controls (Fig. 1C, D). The role of MCU on Bcl-2 was further confirmed in macrophages in vitro. MCU regulated Bcl-2 gene and protein expression (Figs. 1E, F and S1B). Silencing MCU or expression of dominant-negative MCU (MCU_DN_) decreased Bcl-2 expression.

Because MCU increases mitochondrial ROS [23], we determined if MCU regulated *Bcl2* gene expression in a redox-dependent manner. MCU_WT_ increased *Bcl2* mRNA, which was significantly reduced below control levels by MitoTEMPO (Fig. 1G). Antimycin A, a positive control for mitochondrial ROS production, verified the inhibitory effects of MitoTEMPO. The increase in mitochondrial ROS by Antimycin A was decreased below normal by the MitoTEMPO (Fig. S1C). Antimycin A-induced mitochondrial ROS increased *Bcl2* mRNA, while cells treated with MitoTEMPO reduced *Bcl2* gene expression below control levels (Fig. S1D). To determine if Bcl-2 regulated MCU, silencing *Bcl2* had no effect on MCU expression (Fig. S1E) or mitochondrial calcium levels (Fig. S1F). Furthermore, Bcl-2 overexpression did not alter MCU expression (Fig. S1G).

We questioned if the difference in Bcl-2 was functionally relevant. Monocytic cells comprised over 90% of BAL cells from human subjects and mice (Fig. S1H, I). IPF lung macrophages had significantly less TUNEL-positive cells compared to normal subjects (Fig. 1H, I). In vivo, TUNEL-positive lung macrophages from bleomycin-injured *WT* mice were less than the saline controls, while staining in *DN-MCU-Lyz2-cre* mice was significantly greater (Fig. 1J, K).

To determine if the regulation of apoptosis resistance by MCU requires Bcl-2, *Bcl2* was silenced in macrophages expressing MCU_WT_ or MCU_DN_. Caspase-3 activity was decreased by MCU_WT_ and increased by MCU_DN_. Silencing *Bcl2* increased caspase-3 activity in all conditions (Fig. 1L), suggesting that MCU-mediated apoptosis resistance was Bcl-2-dependent. Like MCU_DN_, silencing MCU increased caspase-3 activity (Fig. 1M), active caspase-3 protein, and the pro-apoptotic protein, Bad (Figs. 1N and S1J, K).

Transcriptional repression of anti-apoptotic proteins by p53 induces activation of pro-apoptotic proteins by directly inhibiting Bcl-2 anti-apoptotic function. We found that p53 was markedly increased in the nucleus of lung macrophages from bleomycin-injured *DN-MCU-Lyz2-cre* mice (Fig. S1L). Silencing MCU in macrophages increased p53 content in the nucleus and mitochondria (Fig. S1M), suggesting a direct and indirect regulation of p53 in inducing apoptosis in the absence of MCU in macrophages.

### MCU is associated with apoptosis resistance and inhibition of the mitochondrial intrinsic pathway

**Fig. 2:**
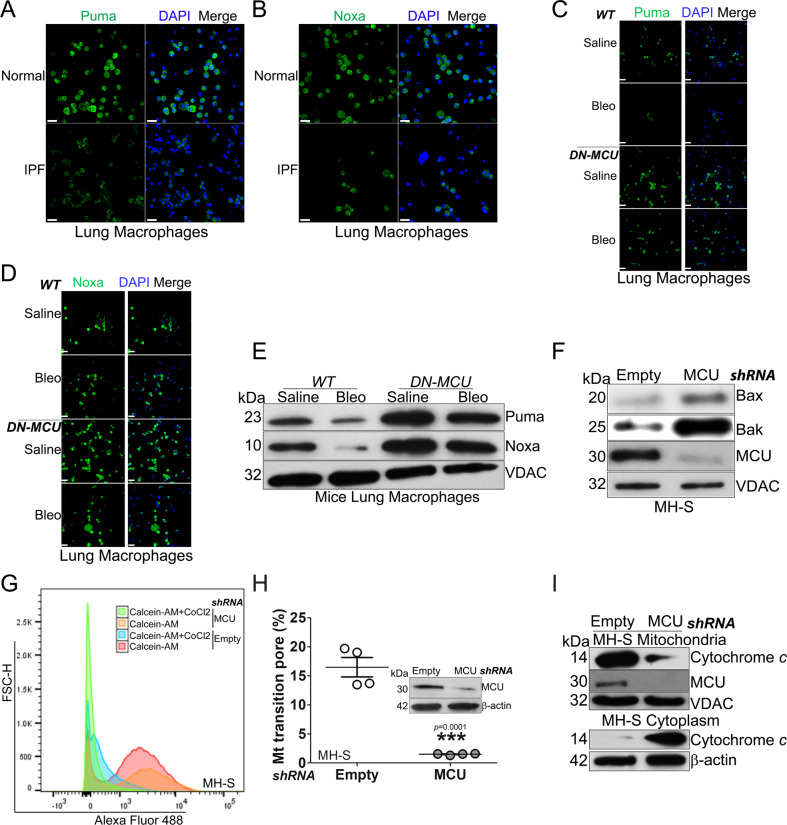
MCU is associated with apoptosis resistance and inhibition of the mitochondrial intrinsic pathway. Lung macrophages from normal or IPF subjects were stained and imaged for **A** Puma or **B** Noxa by confocal microscopy. Scale bars, 20 μm. Lung macrophages from bleomycin- or saline-exposed *DN-MCU-Lyz2-cre* mice or WT littermates were stained and imaged for **C** Puma or **D** Noxa by confocal microscopy. Scale bars, 20 μm. **E** Lung macrophages from *DN-MCU-Lyz2-cre* mice or WT littermates were subjected to mitochondrial isolation and immunoblot analysis for Puma and Noxa. MH-S cells were transfected with empty or MCU shRNA. **F** Immunoblot analysis for Bax and Bak in isolated mitochondria, and **G** mitochondrial permeability transition pore opening were determined in live cells by flow cytometry, and **H** quantified, *n* = 4. **I** Macrophages were transfected with empty or MCU shRNA. Immunoblot analysis for cytochrome *c* was performed in isolated mitochondria and cytoplasm. Two-tailed student’s *t*-test. **p ≤ 0.01. See also Fig. S2.

Based on the increased expression of Bcl-2 in lung macrophages, we asked if other components of the intrinsic pathway were also altered in mediating macrophage apoptosis resistance. The pro-apoptotic proteins in the upstream portion of the intrinsic pathway, Puma (Figs. 2A and S2A) and Noxa (Figs. 2B and S2B), were significantly reduced in lung macrophages from IPF subjects. Similarly, Puma and Noxa expression reduced less than the saline control in macrophages from bleomycin-injured *WT* mice, whereas *DN-MCU-Lyz2-cre* mice had significantly increased content of Noxa and Puma (Fig. 2C, D and Fig. S2C, D). These findings were validated by immunoblot analysis in isolated mitochondria (Fig. 2E). Genetic manipulation showed that silencing *MCU* in macrophages increased levels of Puma and Noxa in isolated mitochondria (S2E–G).

Mitochondrial outer membrane permeabilization, a process whereby Bax and Bak form the outer mitochondrial membrane pores, is a key step in the intrinsic pathway. In macrophages, silencing *MCU* increased both Bax and Bak in mitochondria (Figs. 2F and S2H, I), which led to the permeability transition pore opening (Figs. 2G, H and S2J). Silencing *MCU* also resulted in the release of cytochrome *c* from mitochondria to cytosol (Fig. 2I) and activated caspase-9 rather than caspase-8 in the extrinsic pathway (Fig. S2K). Taken together, these data demonstrate that MCU regulated cell fate in macrophages by targeting the inhibition of pro-apoptotic proteins.

### MCU modulated binding of Cpt1a with Bcl-2 to induce apoptosis resistance

**Fig. 3:**
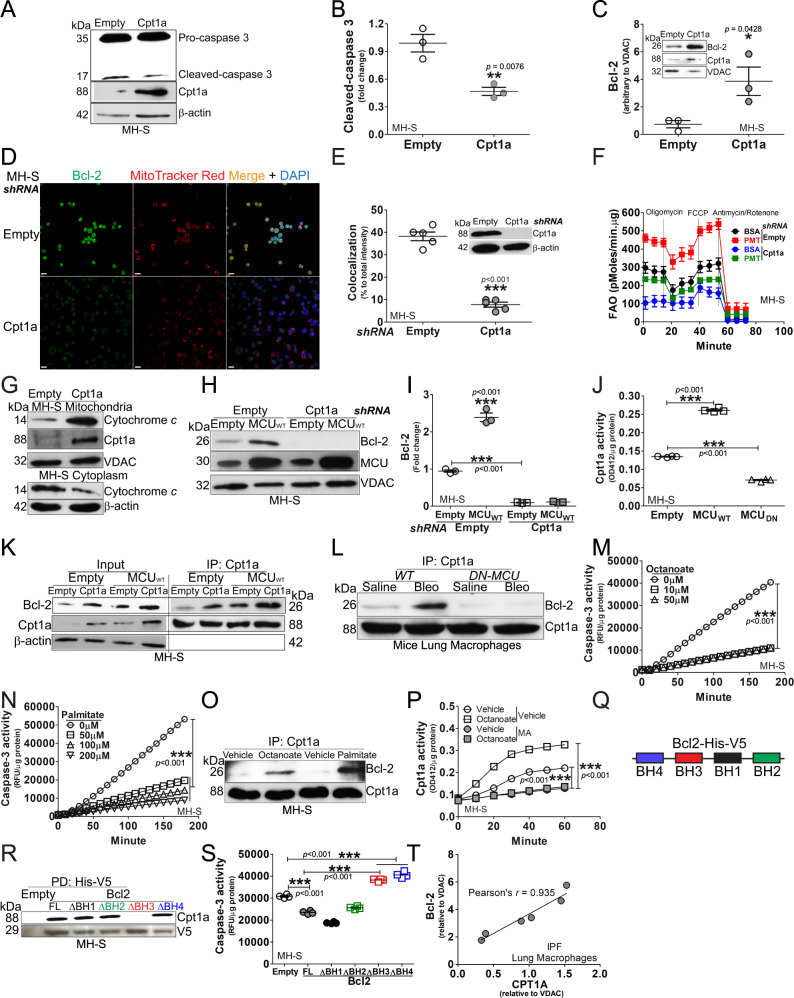
MCU modulated binding of Cpt1a with Bcl-2 to induce apoptosis resistance. MH-S cells were transfected with empty or Cpt1a. Immunoblot analysis for **A** caspase-3 with **B** statistical quantification, *n* = 3, (**C**) Bcl-2 in isolated mitochondria with statistical quantification, *n* = 3. **D** MH-S was transfected with Cpt1a shRNA plasmid or empty vector. Cells were stained with MitoTracker Red and Bcl-2 24 h later and subjected to confocal imaging. **E** The colocalization of Bcl-2 to MitoTracker Red in **D** was quantitated, *n* = 3. **F** MH-S was transfected with Cpt1a shRNA plasmid or empty vector, and cultured for 24 h. Cells were subjected to FAO measurement by Seahorse assay, *n* = 4–6. **G** MH-S was transfected with Cpt1a plasmid or vehicle. Cytochrome *c* in isolated mitochondria and cytoplasm was detected by immunoblot analysis. Macrophages were co-transfected with empty or MCU_WT_ with empty or Cpt1a shRNA. Immunoblot analysis for **H** Bcl-2 with **I** statistical quantification, *n* = 3. **J** MH-S cells were transfected to overexpress MCU_WT_, MCU_DN_, or empty vector. Cpt1a activity was measured, *n* = 4. **K** MH-S cells were co-transfected empty or MCU_WT_ in combination with empty or Cpt1a. Cpt1a was immunoprecipitated and immunoblot analysis for Bcl-2 and Cpt1a was performed. **L** *DN-MCU-Lyz2-cre* mice and *WT* littermates were exposed to saline or bleomycin. Lung macrophages were isolated at 21 days, subjected to Cpt1a immunoprecipitation, and immunoblot analysis for Bcl-2 and Cpt1a. **M** MH-S was treated with octanoate at various concentrations for 3 h. Whole lysate was prepared for determining caspase-3 activities, *n* = 4. **N** MH-S was treated with palmitate at various concentrations for 3 h. Caspase-3 activities were measured, *n* = 4. **O** MH-S was treated with octanoate (10 µM) or palmitate (100 µM) for 3 h. Whole lysate was precipitated with Cpt1a antibody, and elutes were subjected to detection of Bcl-2 and Cpt1a by immunoblot analysis. **P** MH-S was treated with octanoate (10 µM, 4 h), in combination with malonyl CoA (100 µM, 3 h) or vehicle. Cell lysate was prepared for quantitation of Cpt1a activities, *n* = 4. **Q** Schematic of V5-His tagged Bcl-2 with four BH domains. **R** MH-S cells were transfected with Bcl-2-V5-His full length or truncations of BH1, BH2, BH3, or BH4. Bcl-2-V5-His was purified by pull down and immunoblot analysis for Cpt1a was performed. **S** MH-S cells were transfected with empty or Bcl-2-V5-His constructs. Caspase-3 activity was performed, *n* = 4. **T** Pearson’s correlation of CPT1A and Bcl-2 expression in IPF lung macrophages. One-way ANOVA with Tukey’s post hoc comparison (**I**, **J**, **M**, **N**, **S**). Two-tailed student’s *t*-test (**B**, **C**, **E**). *p ≤ 0.05, **p ≤ 0.01, and ***p ≤ 0.001. See also Fig. S3.

Our prior data showed that MCU reprogrammed lung macrophage metabolism to FAO during bleomycin-induced lung fibrosis, in part, by increasing and stabilizing Cpt1a expression and activity [21]. We questioned if Cpt1a had a role in mediating apoptosis resistance in macrophages during fibrosis. Macrophages transfected to increase expression of Cpt1a had a reduction in cleaved caspase-3 (Fig. 3A, B) and increased Bcl-2 in mitochondria (Fig. 3C). Confocal analysis showed that silencing *Cpt1a* in macrophages decreased not only the *Bcl2* expression, but also the Bcl-2 localization to mitochondria (Fig. 3D, E). Silencing *Cpt1a* decreased FAO in macrophages (Fig. 3F), suggesting that the role of Cpt1a in inducing apoptosis resistance may be secondary to its activity in increasing FAO.

Although Cpt1a regulated Bcl-2, overexpression of Bcl-2 (Fig. S3A, B) or silencing *Bcl2* did not modify the expression of Cpt1a in macrophages (Fig. S3C). We further asked if Cpt1a activity contributed to apoptosis resistance. Etomoxir inhibited Cpt1a activity in macrophages (Fig. S3D). The inhibition decreased mitochondrial Bcl-2 (Fig. S3E), increased cleavage in caspase-3 protein (Fig. S3F), and increased caspase-3 activity (Fig. S3G). Etomoxir induced apoptosis regardless of Cpt1a overexpression (Fig. S3H). In aggregate, these results suggest that both Cpt1a expression and activity are required for regulating Bcl-2 expression and resistance to apoptosis.

To determine if Cpt1a attenuated apoptosis via intrinsic pathway, Puma and Noxa localization in mitochondria were decreased in macrophages overexpressing Cpt1a (Fig. S3I). Overexpression of Cpt1a also attenuated the release of cytochrome *c* from mitochondria to cytosol (Fig. 3G).

MCU-mediated apoptosis resistance required Cpt1a. Macrophages expressing MCU_WT_ significantly reduced caspase-3 activity, whereas the inhibition of Cpt1a with etomoxir increased activity above control levels (Fig. S3J). Mitochondrial Bcl-2 expression was increased by MCU_WT_ and decreased below the empty control with etomoxir (Fig. S3K). Silencing *Cpt1a* decreased mitochondrial Bcl-2 below the empty control (Fig. 3H, I).

To determine the relationship between MCU and Cpt1a, we asked if MCU regulated Cpt1a. MCU_WT_ increased Cpt1a activity compared to empty vector, while MCU_DN_ reduced activity below the empty control (Fig. 3J). In contrast, Cpt1a did not regulate MCU expression (Fig. S3L, M). Based on the regulation of Cpt1a activity by MCU, we asked if MCU modulates the binding between Cpt1a and Bcl-2. Cpt1a or MCU_WT_ alone increased Bcl-2 binding, whereas the expression of Cpt1a and MCU_WT_ together increased binding further (Fig. 3K). In vivo, bleomycin-induced injury in *WT* mice increased binding between Cpt1a and Bcl-2 in lung macrophages, whereas the binding was decreased below control levels in *DN-MCU-Lyz2-cre* mice (Fig. 3L).

Because Cpt1a inhibits apoptosis and is the rate-limiting enzyme in FAO, we asked if FAO alone regulated apoptosis. The fatty acid octanoate increased FAO in macrophages, and the addition of palmitate increased OCR further (Fig. S3N). Macrophages treated with octanoate or palmitate showed a significant decrease in caspase-3 activity (Fig. 3M, N), and Bcl-2 expression was increased by either octanoate or palmitate (Fig. S3O, P). Moreover, there was increased binding of Bcl-2 to Cpt1a in macrophages treated with octanoate or palmitate (Fig. 3O).

Malonyl CoA (MA) is known to inhibit Cpt1a activity by disrupting its trimeric structure that is needed for fatty acid transfer into mitochondria [21, 27]. We questioned if the increased binding between Cpt1a and Bcl-2 is attributed to the Cpt1a activity. Octanoate treatment in macrophages significantly increased the Cpt1a activity, which was decreased below control by MA (Fig. 3P). The reduction in Cpt1a activity by MA abrogated the binding between Cpt1a and Bcl-2 (Fig. S3Q).

Because Cpt1a increased localization of Bcl-2 in mitochondria, we asked if there was direct interaction between Cpt1a and Bcl-2 in macrophages during fibrosis. Affinity purification of Cpt1a-His-V5 showed that Bcl-2 was associated with purified Cpt1a (Fig. S3R). To determine if there was specific binding of Cpt1a and Bcl-2, we investigated the four homology (BH) domains within Bcl-2. Full-length Bcl-2 or BH domain truncations fused with a His-V5 tag were generated (Fig. 3Q). Purification of full-length Bcl-2 or the four truncations showed the BH3 truncation prevented binding with Cpt1a (Fig. 3R). The absence of binding of Cpt1a to the truncated BH3 domain had an impact on apoptosis. Caspase-3 activity was decreased below the empty control with full-length Bcl-2 and BH1 and BH2 truncations; however, the activity was increased in truncations of BH3 or BH4 (Fig. 3S). The translational significance showed that CPT1A was highly correlated with Bcl-2 (Pearson’s *r* = 0.935) in IPF lung macrophages (Fig. 3T), suggesting the interaction of these proteins may have a critical role in aberrant fibrotic remodeling. These data also indicate that Cpt1a activity is enhanced during fibrosis that results in increased binding of Bcl-2 in the BH3 domain. In aggregate, these observations suggest a critical link between FAO and apoptosis resistance in lung macrophages.

### Mice harboring a conditional deletion of *Bcl2* in monocyte-derived macrophages are protected from pulmonary fibrosis

**Fig. 4:**
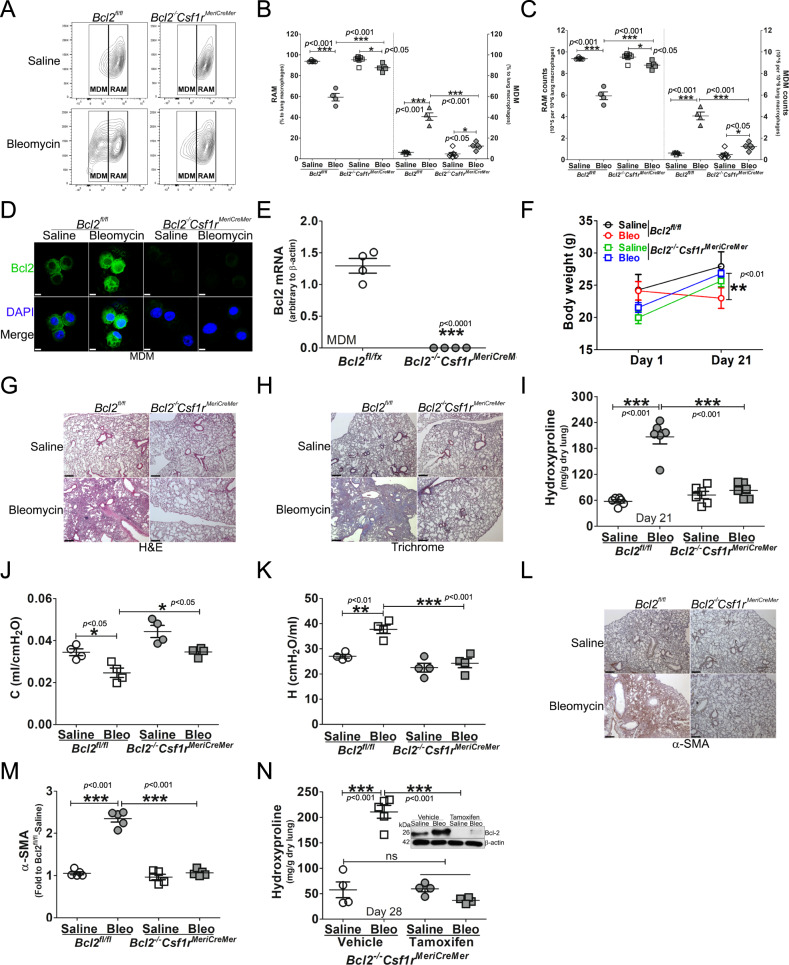
Mice harboring a conditional deletion of *Bcl2* in monocyte-derived macrophages are protected from pulmonary fibrosis. *Bcl2*^*−/−*^*Csf1r*^*MeriCreMer*^ mice and their *Bcl2*^*fl/fl*^ littermates were exposed to saline or bleomycin for 21 days. Lung macrophages from bleomycin- or saline-exposed *Bcl2*^*−/−*^*Csf1r*^*MeriCreMer*^ mice and their *Bcl2*^*fl/fl*^ littermates were **A** analyzed by flow cytometry to distinguish MDM and resident alveolar macrophages (RAM), and statistically quantified by **B** percentage, *n* = 5, or **C** cell counts, *n* = 5. Monocyte-derived macrophages (MDM) from *Bcl2*^*−/−*^*Csf1r*^*MeriCreMer*^ mice and their *Bcl2*^*fl/fl*^ littermates were **D** stained and imaged for Bcl-2 protein by confocal analysis, scale bars at 10 μm, or **E** subjected to total RNA extraction for the detection of *Bcl2* mRNA by qRT-PCR, *n* = 4. **F** *Bcl2*^*−/−*^*Csf1r*^*MeriCreMer*^ mice and their *Bcl2*^*fl/fl*^ littermates were exposed to saline or bleomycin. Mice bodyweights were measured at days 1 and 21 post exposure, *n* = 9–13/group. The lung tissue from bleomycin- or saline-exposed *Bcl2*^*−/−*^*Csf1r*^*MeriCreMer*^ mice and their *Bcl2*^*fl/fl*^ littermates were subjected to **G** H&E staining; **H** Masson’s trichrome staining, represented micrographs from six mice per condition are shown. Scale bars, 200 μm at x5; (**I**) Hydroxyproline, *n* = 6. **J** Compliance (**C**) and **K** tissue stiffness (H) were determined by Respiratory mechanics analysis, *n* = 4. **L** α-SMA measurement by IHC-P and **M** corresponding quantitation of α-SMA, *n* = 5. *Bcl2*^*−/−*^*Csf1r*^*MeriCreMer*^ mice and their *Bcl2*^*fl/fl*^ littermates were exposed to saline or bleomycin for 21 days. **N** *Bcl2*^*−/−*^*Csf1r*^*MeriCreMer*^ mice were exposed to Bleomycin or saline. Mice were intraperitoneally injected daily with corn oil dissolved tamoxifen or corn oil from day 12 until day 20 (20 mg/kg); break per every two injections, and mice were lavaged at day 28. Mice lungs were processed for determination of hydroxyproline content by hydroxyproline assay, *n* = 4. Two-way ANOVA (**F**). One-way ANOVA with Tukey’s post hoc comparison (**B**, **C**, **I**, **K**–**N**). Two-tailed student’s *t*-test (**E**). *p ≤ 0.05, **p ≤ 0.01, and ***p ≤ 0.001. See also Fig. S4.

To determine the effect of the Cpt1a-Bcl-2 interaction in fibrosis development, *Bcl2*^*−/−*^*Csf1r*^*MeriCreMer*^ mice and their control littermates, *Bcl2*^*fl/fl*^ mice, were exposed to saline or bleomycin. Cell differential showed that over 90% of BAL cells were monocytic in both strains (Fig. S4A). Based on the importance of MDMs in fibrosis development [15, 28], the flow cytometry strategy (Fig. S4B) revealed bleomycin-induced injury significantly increased MDMs in *Bcl2*^*fl/fl*^ mice, while decreasing RAMs (Fig. 4A–C). The conditional deletion of *Bcl-2* in mice diminished the increase in MDMs, and RAMs remained increased at the level of the saline control. Similar changes were seen in mice exposed to chrysotile asbestos (Fig. S4C). Bcl-2 was increased in MDMs sorted from bleomycin-injured *Bcl2*^*fl/fl*^ mice and undetectable in the MDMs of *Bcl2*^*−/−*^*Csf1r*^*MeriCreMer*^ mice (Fig. 4D). The absence of Bcl-2 in MDMs of *Bcl2*^*−/−*^*Csf1r*^*MeriCreMer*^ mice was confirmed with qRT-PCR (Fig. 4E).

Bleomycin-induced injury resulted in weight loss in *Bcl2*^*fl/fl*^ mice, while *Bcl2*^*−/−*^*Csf1r*^*MeriCreMer*^ mice gained weight (Fig. 4F). Hematoxylin and Eosin staining showed increased cellular infiltrate and distortion of the lung architecture in *Bcl2*^*fl/fl*^ mice injured with bleomycin, while the *Bcl2*^*−/−*^*Csf1r*^*MeriCreMer*^ mice had normal lung parenchyma (Fig. 4G). Masson’s trichrome staining revealed that deletion of *Bcl2* had no effect on lung parenchyma in saline-exposed mice. There was wide-spread, dense collagen deposition in bleomycin-injured *Bcl2*^*fl/fl*^ mice, while the lungs of *Bcl2*^*−/−*^*Csf1r*^*MeriCreMer*^ mice had no change in collagen expression compared to the saline control mice (Fig. 4H). The histological findings were confirmed biochemically by hydroxyproline assay (Fig. 4I). Correlation of fibrosis with lung function showed that bleomycin decreased compliance and increased tissue stiffness in lungs from *Bcl2*^*fl/fl*^ mice, whereas the conditional deletion of *Bcl2* resulted in reducing the restrictive physiology to the saline controls (Fig. 4J, K). The marker for myofibroblast differentiation, α-SMA, was significantly greater in the lungs of bleomycin-injured *Bcl2*^*fl/fl*^ mice, whereas the expression of α-SMA in the lungs *of Bcl2*^*−/−*^*Csf1r*^*MeriCreMer*^ mice was no different than the saline controls (Fig. 4L, M). Furthermore, *Bcl2*^*−/−*^*Csf1r*^*MeriCreMer*^ mice were also protected from asbestos-induced pulmonary fibrosis (Fig. S4D, E). Taken together, these data strongly suggest that Bcl-2 expression in MDMs has a critical role in fibrosis development.

To directly test the role of Bcl-2 in MDMs during fibrotic progression in mice with established fibrosis, a conditional deletion of *Bcl2* was delayed in *Bcl2*^*−/−*^*Csf1r*^*MeriCreMer*^ mice until day 12 after bleomycin exposure. Hydroxyproline content was significantly greater in the lungs of the *Bcl2*^*−/−*^*Csf1r*^*MeriCreMer*^ mice given vehicle than in mice harboring the *Bcl2* deletion, which was similar to the saline controls (Fig. 4N). These observations suggest that the role of Bcl-2 in MDMs is persistent and is required to mediate dysregulated fibrotic remodeling and fibrosis progression.

### Mice harboring a conditional deletion of *Bcl2* potentiates apoptosis of monocyte-derived macrophages

**Fig. 5:**
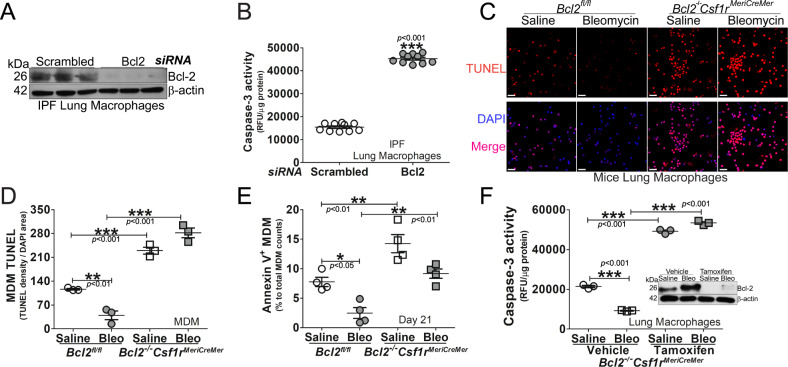
Mice harboring a conditional deletion of *Bcl2* potentiates apoptosis of monocyte-derived macrophages. IPF lung macrophages were transfected with human scrambled or *Bcl2* siRNA. **A** Immunoblot for Bcl-2 and **B** caspase-3 activity, *n* = 10. **C** Lung macrophages from bleomycin- or saline-exposed *Bcl2*^*−/−*^*Csf1r*^*MeriCreMer*^ mice and their *Bcl2*^*fl/fl*^ littermates were stained with TUNEL and imaged by confocal microscopy. Scale bars, 20 μm. Monocyte-derived macrophages (MDM) from bleomycin- or saline-exposed *Bcl2*^*−/−*^*Csf1r*^*MeriCreMer*^ mice and their *Bcl2*^*fl/fl*^ littermates were **D** stained with TUNEL and imaged by confocal microscopy was quantified, *n* = 3, or **E** stained for the detection of Annexin V by flow cytometry, *n* = 4. *Bcl2*^*−/−*^*Csf1r*^*MeriCreMer*^ mice were exposed to Bleomycin or saline. Mice were intraperitoneally injected daily with corn oil dissolved tamoxifen or corn oil from day 12 until day 20 (20 mg/kg; break per every two injections), and mice were lavaged at day 28. **F** BAL macrophages were prepared for detection of caspase-3 activities, *n* = 3, and Bcl-2 by immunoblot analysis. One-way ANOVA with Tukey’s post hoc comparison. Two-tailed student’s *t*-test for **B**. *p ≤ 0.05, **p ≤ 0.01, and ***p ≤ 0.001. See also Fig. S5.

Based on the importance of the mitochondrial localization of Bcl-2 in MDMs for fibrosis development, we asked if the abrogation of apoptosis in IPF lung macrophages required Bcl-2. Silencing *Bcl2* in mitochondria of lung macrophages isolated from IPF subjects (Fig. 5A) significantly increased caspase-3 activity (Fig. 5B). Because *Bcl2*^*−/−*^*Csf1r*^*MeriCreMer*^ mice were protected from bleomycin-induced pulmonary fibrosis, we questioned if the protection was secondary to increased apoptosis in MDMs. There were significantly higher TUNEL-positive lung macrophages from *Bcl2*^*−/−*^*Csf1r*^*MeriCreMer*^ mice compared to the *Bcl2*^*fl/fl*^ mice (Figs. 5C and S5A). Similar TUNEL staining was seen in MDMs (Fig. 5D). To distinguish apoptotic cells from dead cells, Annexin V-positive MDMs were significantly decreased in bleomycin-injured *Bcl2*^*fl/fl*^ mice, while Annexin V was increased in *Bcl2*^*−/−*^*Csf1r*^*MeriCreMer*^ mice (Fig. 5E). The annexin V-positive MDMs had a similar profile in asbestos-exposed mice (Fig. S5B). In established fibrosis, the delay in the conditional deletion of *Bcl2* increased caspase-3 activity in BAL cells (Fig. 5F). These data indicate that Cpt1a-Bcl-2 binding is required for recruitment and apoptosis resistance in MDMs. These observations also indicate a unique relationship between metabolic reprogramming to FAO and apoptosis resistance in fibrosis progression.

### Inhibition of Bcl-2 prevents interaction with Cpt1a and protects mice from fibrosis

To support the requirement of mitochondrial Bcl-2 and Cpt1a-Blc-2 interaction, we used ABT-199, an inhibitor of Bcl-2, to determine if Bcl-2 is a potential therapeutic target to prevent fibrotic remodeling. ABT-199, which is a BH3 mimetic, is known to be effective in hematologic malignancies and skin fibrosis [14, 29, 30]; however, its effect in mitigating apoptosis resistance in MDMs and reversing fibrotic remodeling in the lung is not known.

**Fig. 6:**
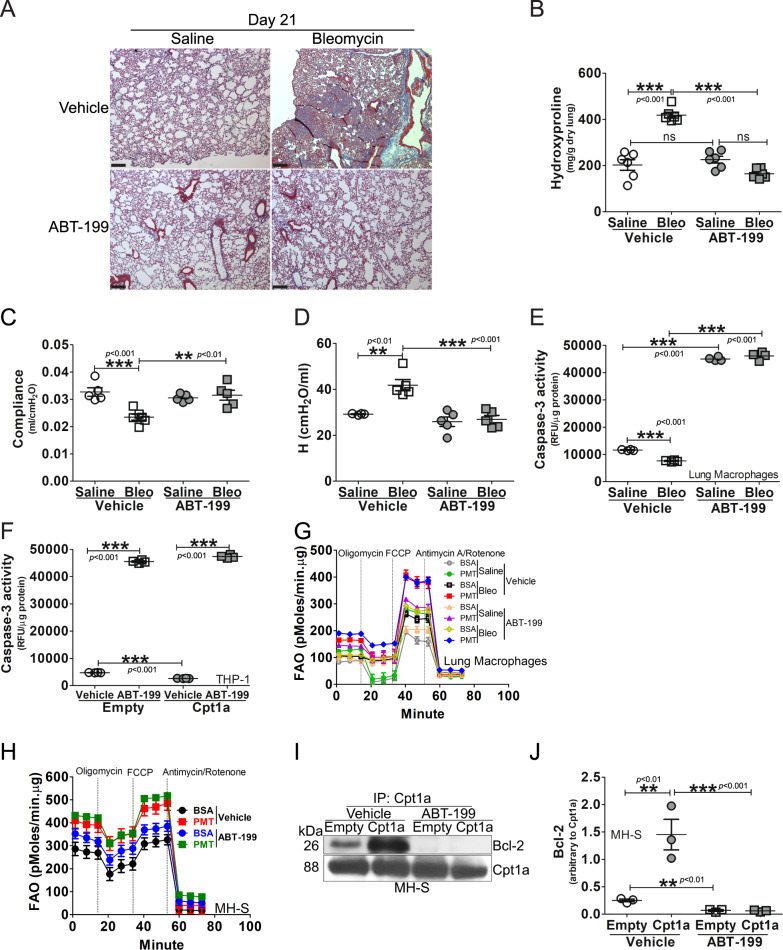
Inhibition of Bcl-2 prevents interaction with Cpt1a and protects mice from fibrosis. *Bcl2*^*fl/fl*^ mice were exposed to saline or bleomycin (Bleo). ABT-199, at the concentration of 50 mg/kg, was administered daily to mice at day 12 days post the exposure until day 21. Lung tissue was subjected to **A** Masson’s trichrome staining, representative micrographs from six mice per condition are shown, with scale bars at 200 μm, x5. **B** Hydroxyproline, *n* = 6. *Bcl2*^*fl/fl*^ mice were exposed to saline or bleomycin (Bleo). ABT-199, at the concentration of 50 mg/kg, was administered daily to mice at day 12 days post the exposure until day 21. **C** Compliance and **D** H stiffness were determined by Respiratory mechanics analysis, *n* = 5. **E** WT mice were exposed to saline or bleomycin (Bleo). ABT-199, at the concentration of 50 mg/kg, was administered daily to mice at day 12 days post the exposure, until day 21. Caspase-3 activity was determined in lavaged lung macrophages, *n* = 4. **F** THP-1 cells were transfected with empty or Cpt1a and treated with ABT-199 (1 μM, overnight) or vehicle. Cells were subjected to quantification of caspase-3 activity, *n* = 4. **G** WT mice were exposed to saline or bleomycin (Bleo). ABT-199, at the concentration of 50 mg/kg, was administered daily to mice at day 12 days post the exposure, until day 21. Fatty acid oxidation of BAL cells was measured by OCR on the Seahorse XF96 bioanalyzer. **H** MH-S was treated with ABT-199 (1 µM, 3 h), and subjected to FAO measurement, *n* = 6. MH-S cells were transfected to overexpress Cpt1a and treated with ABT-199 (1 μM, overnight) or vehicle. Cell lysate was **I** immunoprecipitated with Cpt1a antibody and subjected to immunoblot analysis for Bcl-2, and **J** Bcl-2 was statistically quantitated, *n* = 3. One-way ANOVA with Tukey’s post hoc comparison. **p ≤ 0.01, ***p ≤ 0.001. See also Fig. S6.

We exposed WT mice to saline or bleomycin. On day 12, after fibrosis is established (Fig. S6A), mice were administered vehicle or ABT-199 daily. There was no difference in the BAL cell differential with the majority being a monocytic cell type (Fig. S6B). ABT-199 did not alter the lung parenchyma in the saline-exposed mice (Fig. 6A). Bleomycin-induced injury resulted in wide-spread collagen deposition in the mice that received vehicle, whereas the mice that received ABT-199 had normal lung architecture and no collagen deposition. These histological findings were confirmed biochemically by hydroxyproline assay (Fig. 6B). Inhibition of Bcl-2 with ABT-199 significantly reduced bleomycin-induced restrictive physiology to the vehicle controls (Fig. 6C, D).

To determine if this protection was due to macrophage apoptosis, lung macrophages from mice administered ABT-199 showed a significant increase in caspase-3 activity compared to the saline and bleomycin-injured mice that received vehicle (Fig. 6E). Increased expression of Cpt1a did not alter the effect of ABT-199 inducing apoptosis (Fig. 6F). In vitro, the caspase-3 activity was similar at multiple tenfold concentrations of ABT-199 (Fig. S6C). IHC showed that TUNEL-positive lung fibroblasts were decreased in bleomycin-exposed *Bcl2*^*fl/fl*^ mice when compared to saline (Fig. S6D). TUNEL-positive lung fibroblasts were increased in *Bcl2*^*fl/fl*^ mice with ABT-199 treatment; however, TUNEL staining of fibroblasts from *Bcl2*^*−/−*^*Csf1r*^*MeriCreMer*^ mice that received tamoxifen was similar to the *Bcl2*^*fl/fl*^ mice with vehicle treatment, suggesting that MDM apoptosis has an important role in fibroblast cell fate. The TUNEL staining in fibroblasts from vehicle-treated *Bcl2*^*fl/fl*^ mice and *Bcl2*^*−/−*^*Csf1r*^*MeriCreMer*^ mice was similar after bleomycin injury due the fact that tamoxifen induces *Bcl2* gene depletion only in MDMs rather than fibroblasts.

Because the long-standing dogma indicates that type II AEC injury and subsequent apoptosis are required for the development of lung fibrosis [31, 32], we determined the effect of ABT-199 on AEC. As expected, bleomycin-induced injury increased caspase-3 activity in vehicle-treated mice, and administration of ABT-199 in mice significantly increased the activity further in AECs regardless of the exposure (Fig. S6E). These data suggest that lung macrophages, especially MDMs, have a decisive role in fibrotic remodeling, and induction of macrophage apoptosis with ABT-199 reverses established fibrosis irrespective of AEC apoptosis. These data further suggest that AEC apoptosis, alone, does not induce fibrosis and requires other cells, specifically MDMs, to mediate fibrotic remodeling.

Because Cpt1a is the rate-limiting enzyme for FAO and Cpt1a-Bcl-2 interaction in MDMs is necessary for apoptosis resistance, we questioned if ABT-199-induced inhibition of Bcl-2 altered FAO during fibrosis. Palmitate significantly increased OCR in lung macrophages from bleomycin-injured mice, and ABT-199 did not alter this increase (Figs. 6G and S6F, G). Palmitate increased OCR in all conditions, suggesting that Cpt1a-Bcl-2 interaction does not affect macrophage bioenergetics during fibrosis. These in vivo observations were recapitulated in vitro (Figs. 6H and S6H, I).

Based on the data that ABT-199 increased macrophage apoptosis yet had no effect on metabolic reprogramming to FAO, we asked if ABT-199 inhibited the direct interaction of Cpt1a and Bcl-2. Affinity purification of Cpt1a showed binding to Bcl-2 in vehicle-treated macrophages was greater with Cpt1a overexpression, whereas ABT-199 blocked the Cpt1a-Bcl-2 interaction completely (Fig. 6I, J). These data indicate that MDM apoptosis resistance has a critical role in dysregulated fibrotic remodeling via metabolic reprogramming to FAO from increased Cpt1a activity that results in binding Bcl-2 and anchoring it in the mitochondria. Moreover, induction of macrophage apoptosis by ABT-199 mediated resolution of established fibrosis, suggesting macrophage Bcl-2 is a novel therapeutic target to attenuate fibrotic remodeling in the lung.

### Cpt1a-Bcl-2 binding regulates the macrophage phenotype

**Fig. 7:**
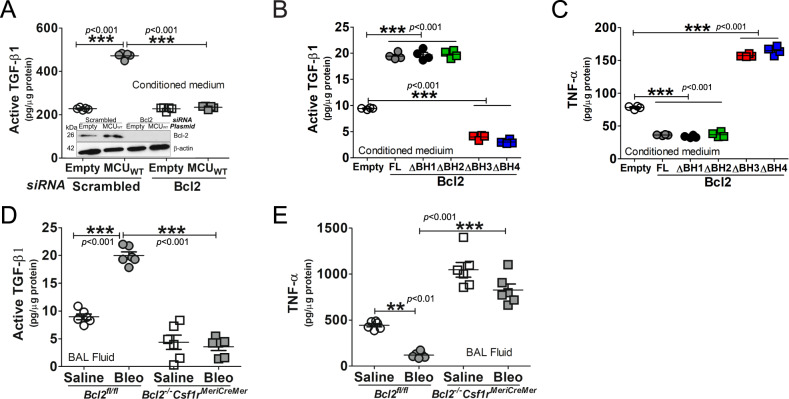
Cpt1a-Bcl-2 binding regulates the macrophage phenotype. **A** MH-S cells were co-transfected with empty or MCU_WT_ and scrambled or Bcl-2 siRNA. Conditioned medium was collected for active TGF-β1 ELISA, *n* = 5. Macrophages were transfected with empty or Bcl-2-V5-His constructs. Conditioned medium was collected for **B** active TGF-β1 or **C** TNF-α by ELISA, *n* = 5. **D** Active TGF-β1 or **E** TNF-α was measured in BAL fluid from bleomycin- or saline-exposed *Bcl2*^*−/−*^*Csf1r*^*MeriCreMer*^ mice and their *Bcl2*^*fl/fl*^ littermates by ELISA, *n* = 5–6/group. One-way ANOVA with Tukey’s post hoc comparison. **p ≤ 0.01 and ***p ≤ 0.001. See also Fig. S7.

Because Bcl-2 binds to Cpt1a to induce apoptosis resistance, we determined whether this interaction modulated macrophage phenotypic polarization. MCU regulated profibrotic polarization of macrophages to increase active TGF-β1 in conditioned media; however, this regulation was reversed when *Bcl2* was silenced (Fig. 7A). Overexpression of the full-length Bcl-2 and the BH1 and BH2 truncations increased the level of active TGF-β1 and decreased the antifibrotic protein, TNF-α, in macrophages (Fig. 7B, C). The expression of the BH3 and BH4 truncations significantly decreased TGF-β1 and increased TNF-α. In vivo, bleomycin-induced injury increased active TGF-β1 and decreased in TNF-α in BAL fluid from *Bcl2*^*fl/fl*^ mice, while an opposite pattern was seen in *Bcl2*^*−/−*^*Csf1r*^*MeriCreMer*^ mice (Fig. 7D, E). Similar changes in active TGF-β1 and TNF-α were observed in asbestos-induced fibrosis (Fig. S7A, B). Taken together, these observations suggest that the direct interaction of Cpt1a and Bcl-2 is a critical determinant of lung macrophage profibrotic polarization, apoptosis resistance, and dysregulated fibrotic remodeling in the lung.

## Discussion

Apoptosis can occur through two distinct pathways, the intrinsic or extrinsic pathway. AECs exhibit both intrinsic and extrinsic pathways of apoptosis during pulmonary fibrosis [32, 33]. Fibroblasts display apoptosis resistance by inhibiting the intrinsic pathway via increased expression of Bcl-2 [13]. The mechanism(s) by which apoptosis resistance occurs in macrophages is associated to the polarization to an anti-inflammatory or alternatively activated phenotype. Prior data demonstrate that lung macrophages are resistant to apoptosis in IPF and in fibrotic mice by the removal of dysfunctional mitochondria by mitophagy [9]. The increase in mitophagy in lung macrophages during fibrosis mediates an increase in macrophage-derived TGF-β1 production. Another mechanism that occurs in atherosclerosis and tissue remodeling is efferocytosis, the engulfment and degradation of apoptotic cells. Efferocytosis reduces inflammation and induces the alternative activation of macrophages [34–36].

**Fig. 8:**
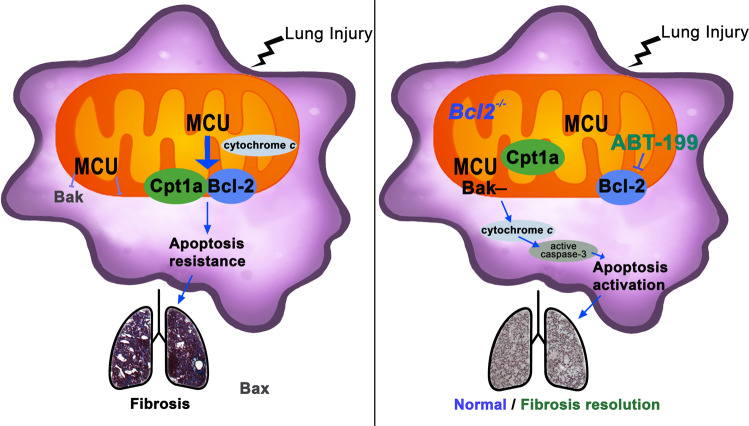
Cpt1a-Bcl-2 binding regulates apoptosis and fibrotic remodeling. Schematic of fibrotic remodeling and apoptosis resistance in monocyte-derived macrophages. MCU increased Cpt1a, which directly binds to Bcl-2 to anchor it in the mitochondria to mediate apoptosis resistance and fibrotic remodeling. Apoptosis activation and resolution of fibrotic remodeling occurred when Bcl-2 was deleted or inhibited with ABT-199 in established fibrosis.

During fibrotic remodeling, MCU mediates metabolic reprogramming to FAO in lung macrophages, in part, by increasing expression and activity of Cpt1a [21]. Cpt1a is the rate-limiting enzyme for FAO that resides in the outer mitochondrial membrane. Cpt1a initiates FAO by converting long-chain acyl-CoA to long-chain acyl-carnitines that are transported into the mitochondria. Previous studies showed the importance of FAO in apoptosis resistance by inhibition of Cpt1a with etomoxir in leukemia and endothelial cells [37, 38]; however, the mechanism by which etomoxir induced apoptosis, other than shifting cellular metabolism to glycolysis, was not determined. Besides its primary function of transporting fatty acids, we show the molecular mechanism by which Cpt1a mediates apoptosis resistance in MDMs by directly binding to Bcl-2, which increased its mitochondrial localization. Our data further demonstrated that MCU was a crucial regulator of this process. MCU regulates *Bcl2* transcriptionally in a redox-dependent manner. *Bcl2* transcription is dependent on the redox-regulated transcription factor NF-κB [39, 40]. Because MCU increases mitochondrial ROS in macrophages during fibrosis [23], this suggests a mechanism by which MCU mediates Cpt1a-Bcl-2 binding and apoptosis resistance (Fig. 8).

Bcl-2 localizes to the mitochondria, endoplasmic reticulum, nuclear envelope, or cytosol, depending on physiological or pathological conditions. The binding of Cpt1a to Bcl-2 anchored Bcl-2 in the mitochondria. Previous studies showed that Bcl-2 binds pro-apoptotic proteins, such as Puma, Bak, or Bad, through the BH3 domain [41, 42]. The mechanism(s) by which Bcl-2 preferentially binds to Cpt1a or to pro-apoptotic proteins is not known. The mitochondrial membrane plays an active role in most Bcl-2 family interactions by changing the affinities and the relative abundance of these proteins to the mitochondrial membrane [43–45].

The investigation of Bcl-2 family proteins has primarily been in cancer and autoimmune disease [30, 46]. Besides domains 1–3, the BH4 domain is also important for the anti-apoptotic activity of Bcl-2, as the BH4 domain alone can prevent apoptotic cell death and apoptotic changes of isolated mitochondria [47]. The BH4 domain inhibits apoptosis by binding the regulatory and coupling domain of the IP3 receptor to regulate calcium release from the endoplasmic reticulum. The deletion of the BH4 domain converts Bcl-2 into a pro-apoptotic protein [48]. The importance of BH4 domain has been shown to be crucial for angiogenesis, vascularization, and metastasis [49]. Moreover, the BH4 domain has been investigated as a therapeutic target in cancer. BDA-366, a potent antagonist of BH4 domain, induces apoptosis in a variety of cancer cell models, and has shown utility in suppressing lung cancer growth [36]. The direct interaction of Cpt1a in the BH3 domain decreased caspase-3 activity in the absence of the BH1 or BH2 domain, but not in the absence of BH4. These data suggest that although Cpt1a binds to the BH3 domain, the function of Bcl-2 requires the BH4 domain as well.

A long-standing belief is that AEC apoptosis is required for the development of lung fibrosis [31, 32, 50]. We have shown that the augmented polarization of MDMs to a profibrotic phenotype mediates fibrosis without AEC injury or apoptosis [16]. Studies in IPF and other forms of pulmonary fibrosis have focused on the role of AECs and fibroblasts as a key feature in the disease pathogenesis [12–14, 32]. Our data show the Bcl-2 inhibitor ABT-199 mediated greater apoptosis of AECs; however, established fibrosis was reversed by ABT-199 secondary to induction of lung macrophage, and potentially fibroblast, apoptosis. Our data also demonstrates that mice harboring a conditional deletion Bcl-2 in MDMs after fibrosis is established have reversal of the dysregulated fibrotic remodeling in the lung. Taken together, these observations suggest that FAO provokes apoptosis resistance through the stabilization of Bcl-2 in the mitochondria by binding to Cpt1a. Moreover, these data demonstrate that MDMs are required for fibrosis progression and suggest a novel therapeutic target to prevent progressive aberrant fibrotic remodeling.

## Supplementary information

Supplemental Material

Figure S1

Figure S2

Figure S3

Figure S4

Figure S5

Figure S6

Figure S7
