# Supplementary material for: Targeting Cpt1a-Bcl-2 interaction modulates apoptosis resistance and fibrotic remodeling
Source: Cell Death Differ. 2021 Aug 20;29(1):118–32. doi: 10.1038/s41418-021-00840-w (PMC8738732; doi:10.1038/s41418-021-00840-w)
Supplement: Supplementary file 1 — Supplemental Material [file 41418_2021_840_MOESM1_ESM.docx]

**Figure legends**

**Figure S1. Macrophage MCU modulates apoptosis resistance of lung macrophages in pulmonary fibrosis.**

(A) The detection of Bcl-2 in figure 1A was quantitated, normal *n* =5, IPF, *n* = 6. (B) The detection of Bcl-2 in figure 1F was quantitated, *n* = 3. (C) THP-1 cells were treated with Antimycin A (50µM, 3 hours), in combination with MitoTEMPO (50µM, 3 hours) or vehicle. Mitochondria were isolated for the measurement of H_2_O_2_ by pHPA assay, *n* = 4. (D) MH-S cells were treated with Antimycin A (50µM, 3 hours), in combination with MitoTEMPO (50µM, 3 hours) or vehicle. Total RNA was extracted for the quantitation of Bcl2 mRNA, *n* = 4. MH-S cells were transfected to silence Bcl2, (E) whole lysate was prepared for detection of MCU by immunoblot analysis, *n* = 3; or (F) Calcium was determined in isolated mitochondria, *n* = 4. (G) MH-S cells were transfected to overexpress Bcl-2. Whole lysate was prepared for detection of MCU by immunoblot analysis, *n* = 3. (H) BAL cell differential from IPF (*n* = 4) and normal (*n* = 5) subjects. Mac: macrophages; Lym: lymphocytes; PMN: polymorphonuclear leukocytes. (I) Statistical quantitation of cell differential in BAL cells from mice (*WT* and *DN-MCU-Lyz2-cre*), *n* = 9~10. (J) The detection of cleaved-caspase 3 in figure 1N was quantitated, *n* = 3. (K) The detection of Bad in figure 1N was quantitated, *n* = 3. (L) Lung macrophages from bleomycin- or saline-exposed *DN-MCU-Lyz2-cre* mice or WT littermates were subjected to nucleus isolation, for the detection of p53 by immunoblot analysis. (M) Macrophages were transfected to silence MCU. Mitochondria and nucleus were isolated for the detection of p53. One-way ANOVA with Tukey's post hoc comparison (B-D, H-I). Two-tailed student's *t*-test. **p* ≤ 0.05, ***p* ≤ 0.01 and ****p* ≤ 0.001.

**Figure S2. MCU modulates apoptosis resistance by inhibiting the mitochondrial intrinsic apoptotic pathway.**

(A) The detection of Puma protein in figure 2A was quantitated, *n* = 5. (B) The detection of Noxa in figure 2B was quantitated, *n* = 5. (C) The detection of Puma in figuer 2C was quantitated, *n* = 3. (D) The detection of Noxa in figure 2D was quantitated, *n* = 3. MH-S cells were transected to silence MCU. Mitochondria were isolated for (E) the detection of Puma and Noxa by immunoblot analysis, and (F) quantitation of Puma, *n* = 3, and (G) quantitation of Noxa, *n* = 3. (H) The detection of Bak in figure 2F was quantitated, *n* = 3. (I) The detection of Bax in figure 2F was quantitated, *n* = 3. (J) MH-S was transfected with empty or MCU shRNA. Mitochondrial permeability transition pore opening was determined in live cells by flow cytometry. (K) MH-S was transfected to silence MCU. Cleaved-caspase 9 and cleaved-caspase 8 were detected by immunoblot analysis. One-way ANOVA with Tukey's post hoc comparison (C and D). Two-tailed student's *t*-test. ***p* ≤ 0.01 and ****p* ≤ 0.001.

**Figure S3. MCU modulated binding of Bcl-2 with Cpt1a to induce apoptosis resistance.**

1. MH-S was transfected with Bcl2 plasmid or empty vector, and cultured for 24 hours. Mitochondria were isolated for detection of Cpt1a by immunoblot analysis, *n* =3. (B) MH-S was transfected to overexpress Bcl-2. Cells were then stained and imaged for the detection of Bcl-2 and Cpt1a by confocal analysis. Scale bars, 20μm. (C) Macrophages were transfected with Bcl2 siRNA or scramble. Cells were subjected to mitochondrial isolation after 24 hours, and subsequent immunoblot detection of Cpt1a and Bcl-2 proteins. MH-S was treated overnight with etomoxir at various concentrations. Cell lysate was prepared for the detection of (D) Cpt1a activities, *n* = 4, (E) Bcl-2 protein, (F) cleaved caspase 3 protein, and (G) caspase 3 activities, n = 4. (H) MH-S was transfected to overexpress Cpt1a and treated with etomoxir (10µM, overnight). Whole lysate was prepared for detection of cleaved caspase 3. (I) MH-S was transfected to overexpress Cpt1a. Mitochondria were isolated for the detection of Puma and Noxa by immunoblot analysis. MH-S was transfected to overexpress MCU_WT_, and treated with etomoxir (10μM, overnight). Cells were (J) lysed for the quantitation of caspase-3 activities, *n* = 4; or (K) subjected to mitochondrial isolation for the detection of Bcl-2 by immunoblot analysis. (L) MH-S was transfected to silence Cpt1a. Whole lysate was prepared for detection of MCU by immunoblot analysis, *n* = 3. (M) MH-S was transfected to overexpress Cpt1a. Whole lysate was prepared for detection of MCU, *n* = 3. (N) MH-S was treated with octanoate (10µM) for 3 hours, and subjected to FAO measurement by seahorse assay, *n* = 4. (O) MH-S was treated with octanoate for 3 hours. Whole lysate was prepared for detection of Bcl-2. (P) MH-S was treated with palmitate for 3 horus. Whole lysate was prepared for detection of Bcl-2. (Q) MH-S was treated with octanoate (10µM, 4 hours), in combination with malonyl CoA (100µM, 3 hours) or vehicle. Cell lysate was subjected to immunoprecipitation by Cpt1a antibody and immunoblot analysis for Bcl-2. (R) THP-1 was transfected to overexpress Cpt1a-His-V5. Cell lysate was precipitated by His-V5 beads, and elutes were subjected to Bcl-2 detection by immunoblot analysis. One-way ANOVA with Tukey's *post hoc* comparison. Two-tailed student's t-test (A, L-M). **p* ≤ 0.05, ***p* ≤ 0.01 and ****p* ≤ 0.001.

**Figure S4. Mice harboring a conditional deletion of Bcl2 in macrophages are protected from pulmonary fibrosis.**

1. *Bcl2^-/-^Csf1r^MeriCreMer^* mice and their *Bcl2^fl/fl^* littermates were exposed to saline or bleomycin. BAL was performed at 21 days. Statistical quantification of cell differential in BAL cells, *n* = 6. Mac: macrophages; Lym: lymphocytes; PMN: polymorphonuclear leukocytes. (B) Gating strategy of sorting monocyte-drived macrophages (MDM) and resident alveolar macrophages (RAM) from BAL cells. (C) *Bcl2^-/-^Csf1r^MeriCreMer^* mice and their *Bcl2^fl/fl^* littermates were exposed to asbestos or MMVF for 21 days. Lung macrophages were analyzed by flow cytometry to determine the level of monocyte-derived macrophages (MDM), *n* = 5. The lung tissue from asbestos- or MMVF-exposed *Bcl2^-/-^Csf1r^MeriCreMer^* mice and their *Bcl2^fl/fl^* littermates were subjected to (D) Masson’s trichrome blue staining, represented micrographs from 6 mice per condition are shown. Scale bars, 200 μm at x5, or (E) quantitation of hydroxyproline, *n* = 5. One-way ANOVA with Tukey's *post hoc* comparison. **p* ≤ 0.05, ***p* ≤ 0.01 and ****p* ≤ 0.001.

**Figure S5. Mice harboring a conditional deletion of *Bcl2* potentiates apoptosis of MDMs.**

(A) The TUNEL detection in figure 5C was quantitated, *n* = 4. (B) Monocyte-derived macrophages (MDM) from asbestos- or MMVF-exposed *Bcl2^-/-^Csf1r^MeriCreMer^* mice and their *Bcl2^fl/fl^* littermates were stained for the detection of Annexin V by flow cytometry, *n* = 5. One-way ANOVA with Tukey’s post hoc comparison. *** *p* ≤ 0.001.

**Figure S6. Inhibition of Bcl-2 prevents interaction with Cpt1a and protects mice from fibrosis.**

(A) *Bcl2^fl/fl^* mice were exposed to saline or bleomycin (bleo) for 12 days. Lung tissue was subjected to Masson’s trichrome blue staining. Represented micrographs from 3 mice per condition are shown. Scale bars, 200 μm at x5. (B) Statistical quantitation of cell differential in BAL cells, n = 6. Mac: macrophages; Lym: lymphocytes; PMN: polymorphonuclear leukocytes. (C) THP-1 cells were treated with various concentration of ABT-199 for overnight. Cells were subjected to measurement of caspase-3 activities, *n* = 4. (D) Statistical analysis to the IHC-P assay detecting α-SMA, Vimentin, and DAPI in mice lung tissues (imaging not shown), *n* = 3. (E) WT mice were exposed to saline or bleomycin (bleo). ABT-199, at the concentration of 50mg.kg, was administered daily to mice at day 12 days post the exposure, until day 21. Type II AEC cells were prepared from lung tissue for the determination of caspase-3 activities, *n* = 4. Fatty acid oxidation by seahorse assay in figure 6G was represented with (F) basal OCR, *n* = 3, and (G) max OCR, *n* = 3. FAO by seahorse assay in figure 6H was represented with (H) basal OCR, *n* = 6, and (I) max OCR, *n* = 6. One-way ANOVA with Tukey's post hoc comparison. ***p* ≤ 0.01, ****p* ≤ 0.001.

**Figure S7. Cpt1a-Bcl-2 binding regulates the macrophage phenotype**

The BAL fluid form asbestos- or MMVF-exposed *Bcl2^-/-^Csf1r^MeriCreMer^* mice and their *Bcl2^fl/fl^* littermates were subjected to detection of (A) active TGF-β1 or (B) TNF-α by cytokine ELISA, *n* = 5. One-way ANOVA with Tukey's post hoc comparison. ****p* ≤ 0.001.
