# Supplementary figures and images for: Targeting Cpt1a-Bcl-2 interaction modulates apoptosis resistance and fibrotic remodeling
Source: Cell Death Differ. 2021 Aug 20;29(1):118–32. doi: 10.1038/s41418-021-00840-w (PMC8738732; doi:10.1038/s41418-021-00840-w)

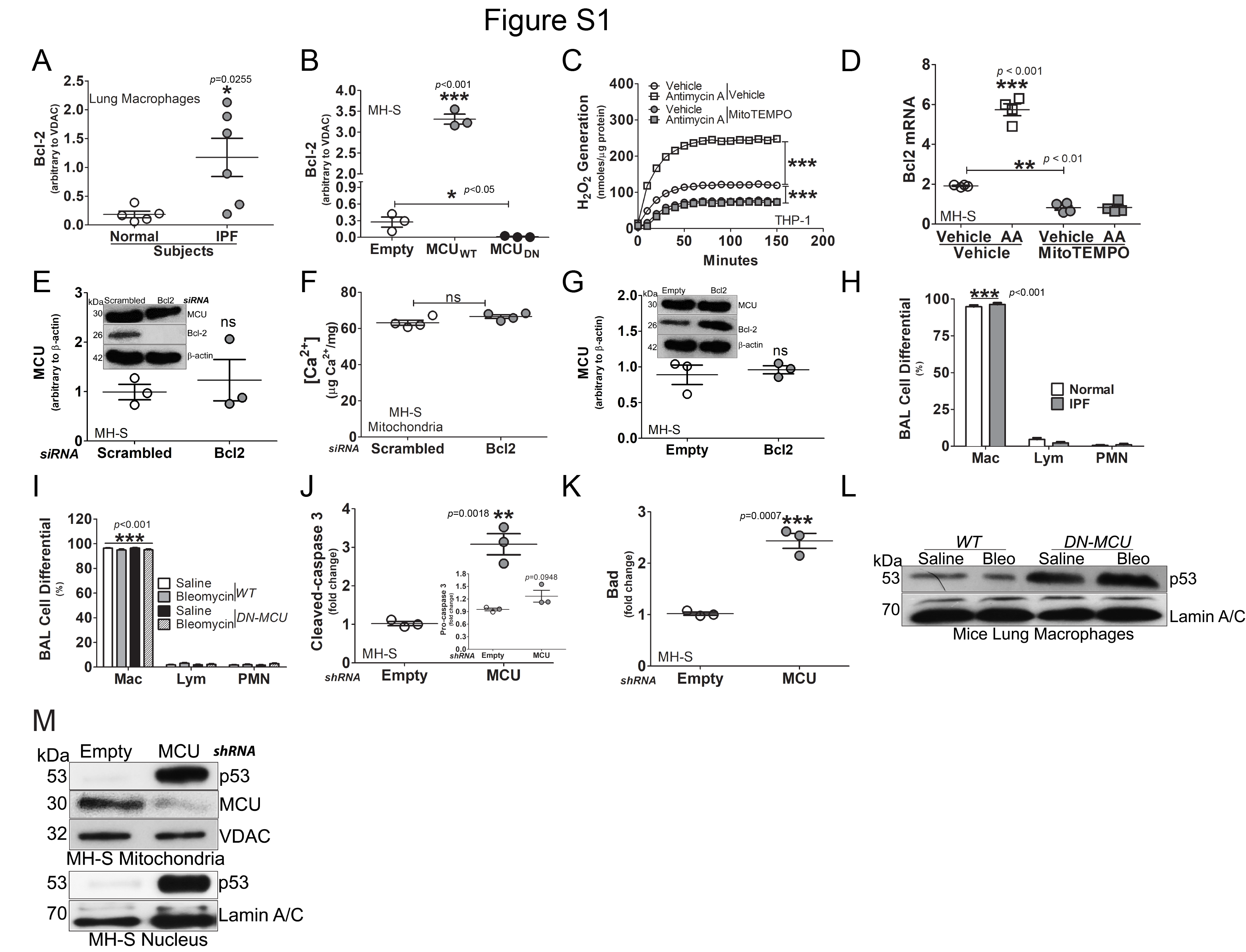

Supplement: Supplementary file 2 — Figure S1 [file 41418_2021_840_MOESM2_ESM.tif]

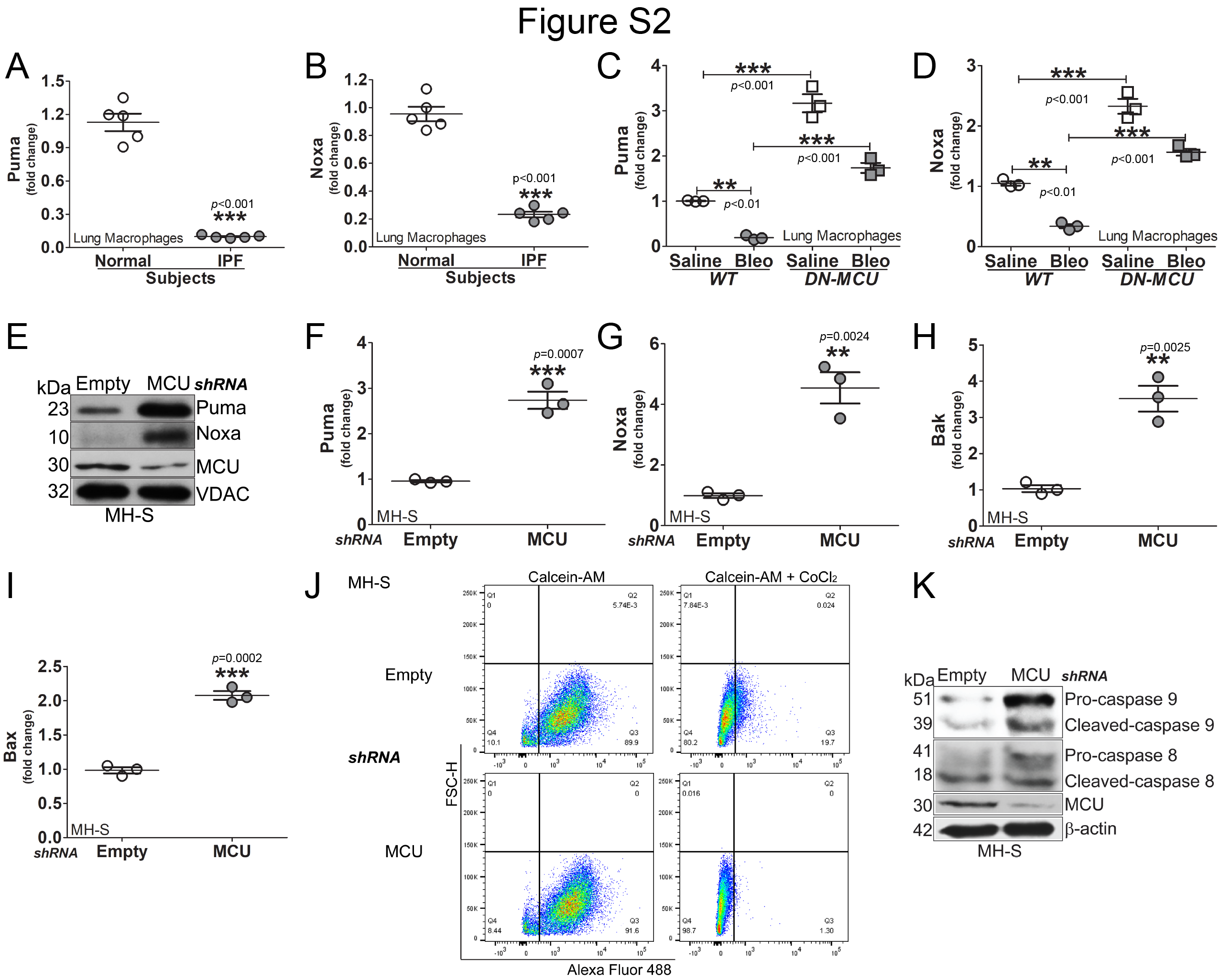

Supplement: Supplementary file 3 — Figure S2 [file 41418_2021_840_MOESM3_ESM.tif]

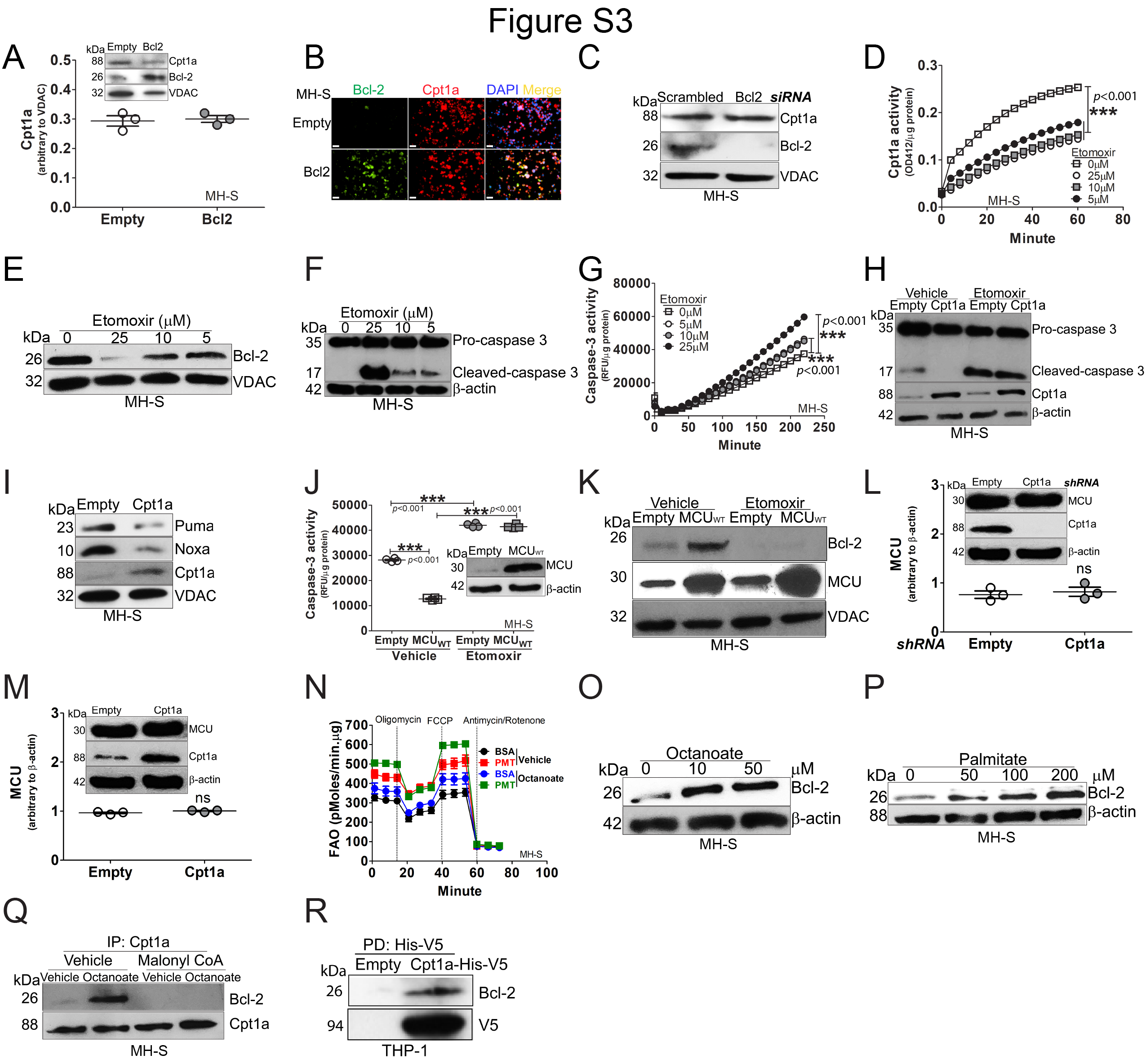

Supplement: Supplementary file 4 — Figure S3 [file 41418_2021_840_MOESM4_ESM.tif]

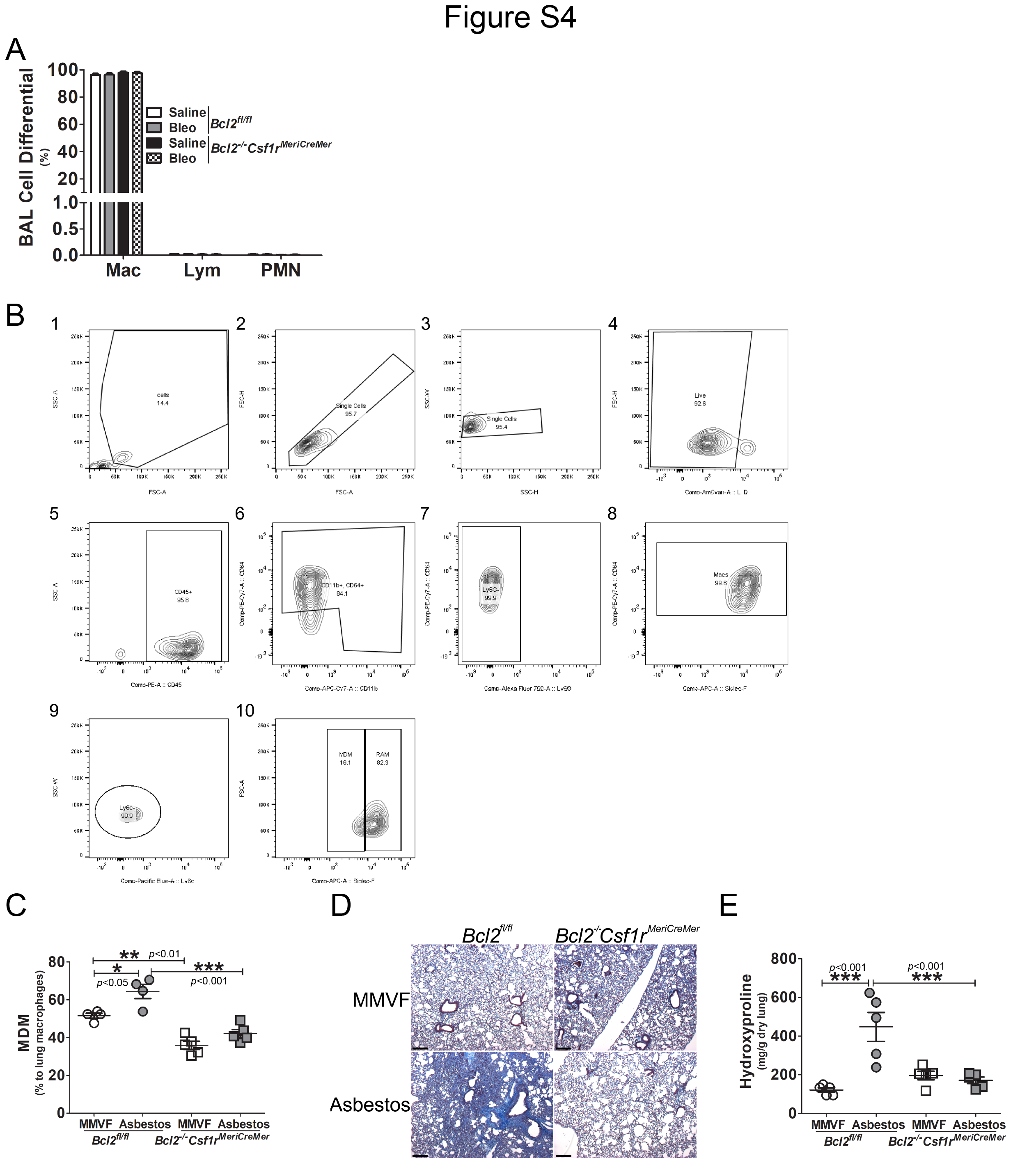

Supplement: Supplementary file 5 — Figure S4 [file 41418_2021_840_MOESM5_ESM.tif]

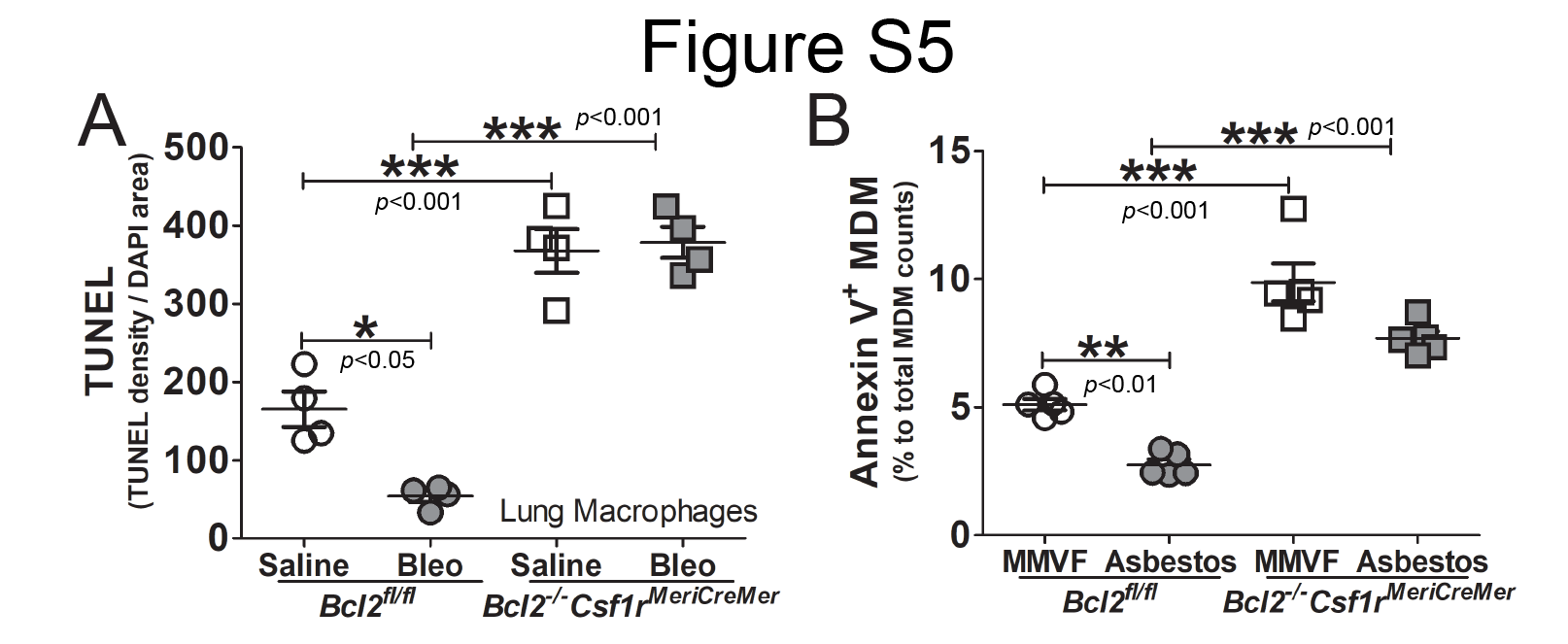

Supplement: Supplementary file 6 — Figure S5 [file 41418_2021_840_MOESM6_ESM.tif]

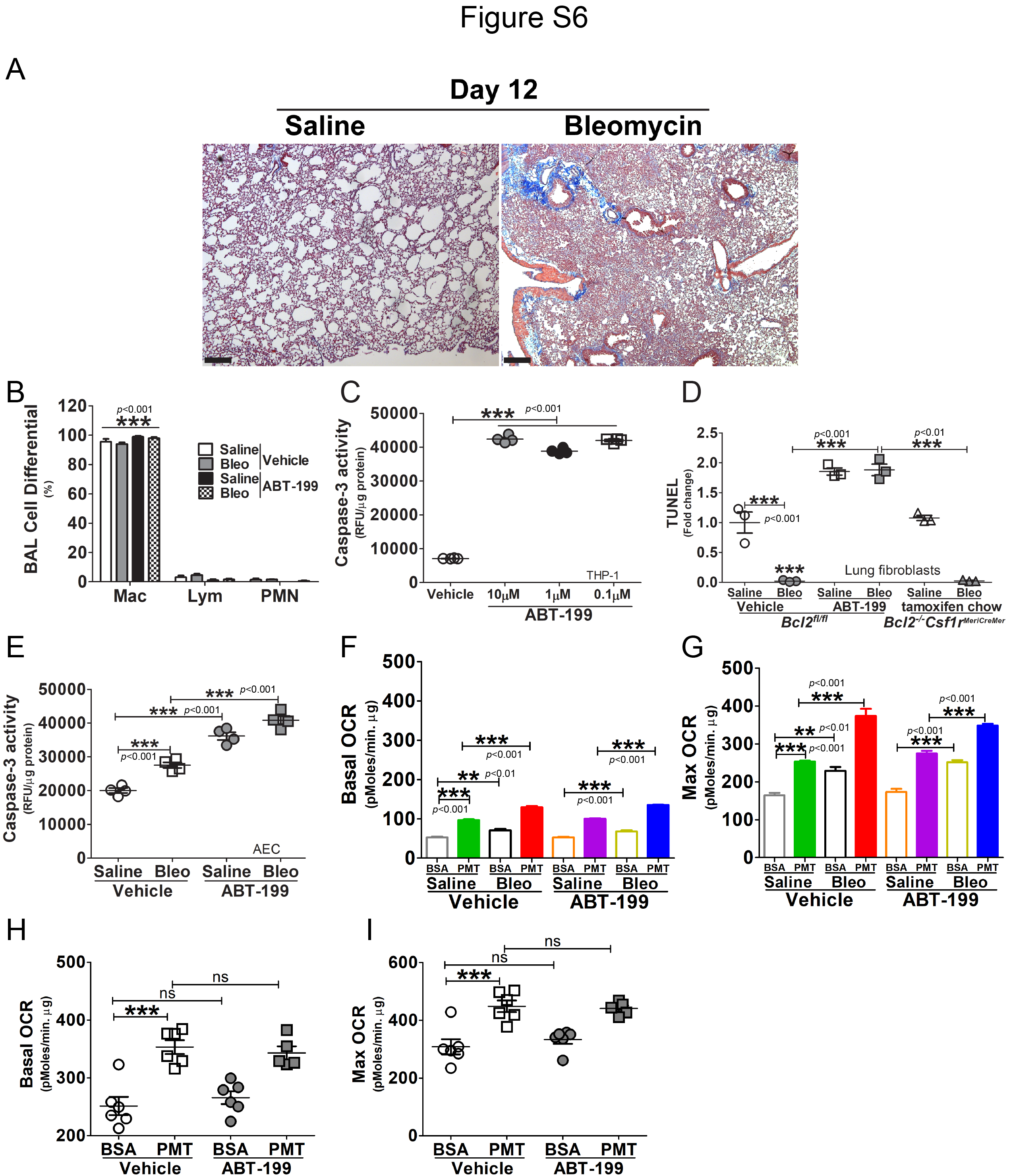

Supplement: Supplementary file 7 — Figure S6 [file 41418_2021_840_MOESM7_ESM.tif]

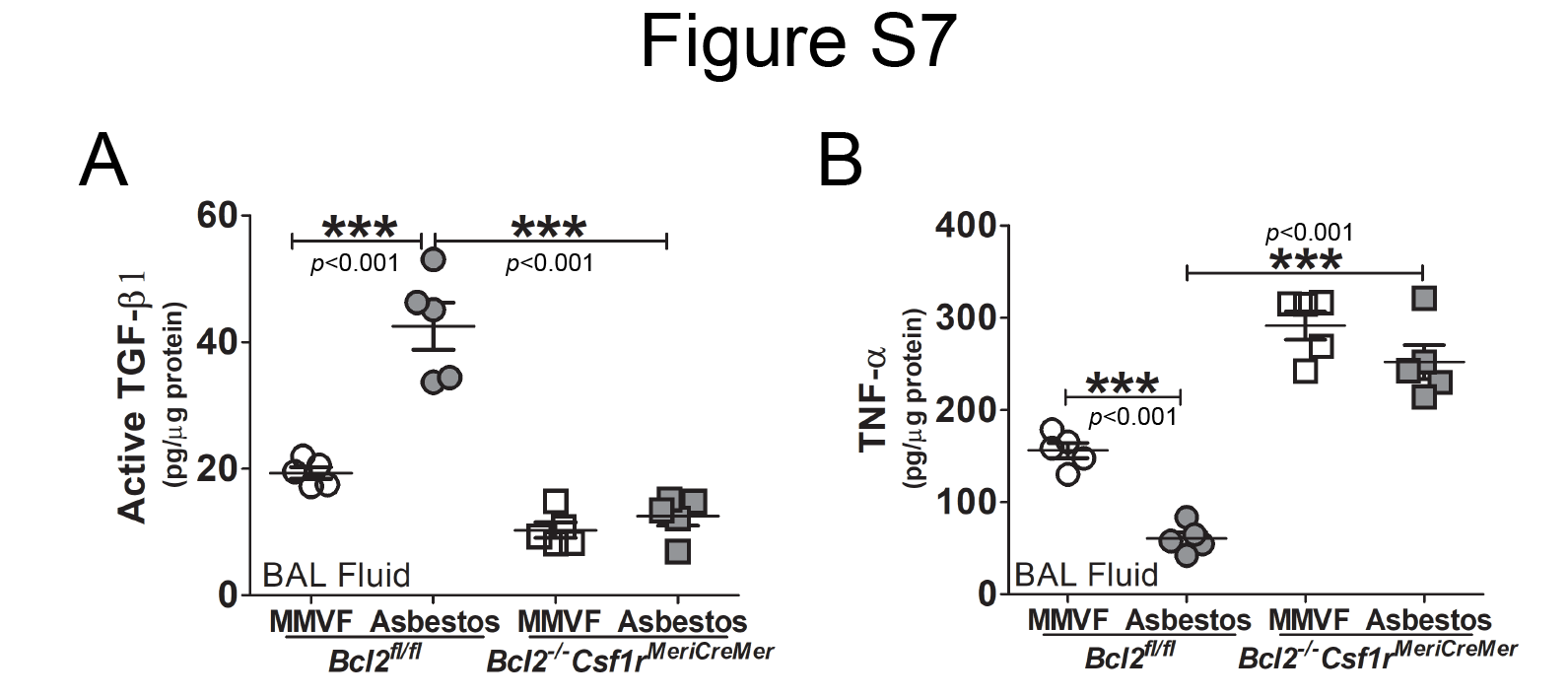

Supplement: Supplementary file 8 — Figure S7 [file 41418_2021_840_MOESM8_ESM.tif]
